# Transcriptomic profiling of blood from autoimmune hepatitis patients reveals potential mechanisms with implications for management

**DOI:** 10.1371/journal.pone.0264307

**Published:** 2022-03-21

**Authors:** Michele May-Sien Tana, Arielle Klepper, Amy Lyden, Angela Oliveira Pisco, Maira Phelps, Breann McGee, Kelsey Green, Sandy Feng, Joseph DeRisi, Emily Dawn Crawford, Craig S. Lammert

**Affiliations:** 1 University of California, San Francisco, CA, United States of America; 2 UCSF Liver Center, San Francisco, CA, United States of America; 3 Chan Zuckerberg Biohub, San Francisco, CA, United States of America; 4 University of Indiana, Bloomington, IN, United States of America; Centro Cardiologico Monzino, ITALY

## Abstract

Autoimmune hepatitis (AIH) is a poorly understood, chronic disease, for which corticosteroids are still the mainstay of therapy and most patients undergo liver biopsy to obtain a diagnosis. We aimed to determine if there was a transcriptomic signature of AIH in the peripheral blood and investigate underlying biologic pathways revealed by gene expression analysis. Whole blood RNA from 75 AIH patients and 25 healthy volunteers was extracted and sequenced. Differential gene expression analysis revealed 249 genes that were significantly differentially expressed in AIH patients compared to controls. Using a random forest algorithm, we determined that less than 10 genes were sufficient to differentiate the two groups in our cohort. Interferon signaling was more active in AIH samples compared to controls, regardless of treatment status. Pegivirus sequences were detected in five AIH samples and 1 healthy sample. The gene expression data and clinical metadata were used to determine 12 genes that were significantly associated with advanced fibrosis in AIH. AIH patients with a partial response to therapy demonstrated decreased evidence of a CD8+ T cell gene expression signal. These findings represent progress in understanding a disease in need of better tests, therapies, and biomarkers.

## Introduction

Autoimmune hepatitis (AIH) is an uncommon, chronic inflammatory disorder of the liver that can lead to cirrhosis, liver transplantation, and death. Epidemiologic studies suggest its incidence and prevalence are rising, and our prior work has shown that AIH disproportionately affects people of color in the United States [1, 2]. It is well-recognized that AIH patients are predominantly female. The pathogenesis of AIH remains obscure and may be influenced by genetic predisposition and unknown environmental triggers, potentially viruses, chemicals, or medications. The result is a dysregulated innate and adaptive immune response, with T- and B-cell mediated inflammation. Normal regulatory mechanisms do not maintain tolerance to as yet unknown autoantigens. There is no single, reliable blood test for the diagnosis of AIH, and an invasive liver biopsy is often required to secure the diagnosis and stage disease severity. Management frequently consists of lifelong nonspecific immunosuppression with azathioprine or other salvage therapies [3].

RNA sequencing (RNA-seq) is a powerful and comprehensive tool that provides rapid, affordable, and high-resolution analysis of transcriptomes. RNA-seq has diverse applications across multiple fields of biology and can be used to assess transcriptomic profiles, discover novel biomarkers, and evaluate pathophysiologic mechanisms. Multiple studies have analyzed cytokines of interest and specific lymphocyte populations in patients with AIH, but no research has focused on an unbiased look at gene expression in the peripheral blood.

In this study, we performed whole blood transcriptomic analysis using RNA-Seq to determine whether peripheral blood gene expression signatures could distinguish AIH patients from healthy controls, and to explore biologic pathways underlying AIH. Our goals were to identify signatures that may reflect clinical markers of AIH disease activity and shed light on the pathophysiologic basis of this enigmatic disease.

## Methods

### Clinical cohort

This work was approved by the institutional review boards at Indiana University and University of California, San Francisco. Data were analyzed in a deidentified manner. RNA was provided by the Genetic Repository of Autoimmune Liver Diseases and Contributing Exposures (GRACE) cohort, established in 2014 at Indiana University [4]. This cohort was developed to strategically warehouse and link biospecimens, high quality clinical data, and environmental exposure histories among autoimmune liver disease patients [5]. AIH patients (cases) included in the GRACE cohort included adults with a diagnosis of AIH meeting at least the simplified AIH criteria [5, 6]. Cases meeting inclusion criteria were serially recruited from in- and outpatient settings. Controls were healthy volunteers, recruited from the community at Autoimmune Hepatitis Association conferences and other events. They were screened for liver disease through self-reported history and through blood tests of liver enzymes. All participants provided informed consent.

Clinical data collected included demographics, time of diagnosis and biospecimen collection, medications, laboratory results, imaging studies, liver histology, and outcomes such as decompensation. Study data were collected and managed using REDCap electronic data capture tools hosted at UCSF [7]. REDCap (Research Electronic Data Capture) is a secure, web-based software platform designed to support data capture for research studies, providing 1) an intuitive interface for validated data capture; 2) audit trails for tracking data manipulation and export procedures; 3) automated export procedures for seamless data downloads to common statistical packages; and 4) procedures for data integration and interoperability with external sources.

Autoimmune hepatitis type was determined through chart review. Type 1 was defined by positivity of antinuclear, anti-smooth muscle, or anti-actin antibodies. Type 2 was defined by antibodies to LKM-1 or LC-1. Cases following exposure to an offending drug with the ability to wean immunosuppression was classified as drug-induced AIH. Because not every patient had a liver biopsy near the time for blood collection, we use the following algorithm to estimate fibrosis stage (S1 Fig). Fibrosis stage was based on liver biopsy (preferred) or transient elastography within six months, either before or after, specimen collection. Patients with values > 19kPa were deemed cirrhotic and those with values < 13 non-cirrhotic. For TE values between 13 and 19, FIB-4 was calculated [8]. When neither biopsy nor TE were available, then FIB-4 value < 2.6 was interpreted as non-cirrhotic [9]. For patients with FIB-4 values > = 2.6, results of ultrasound, computed tomography, or magnetic resonance imaging were reviewed for corroborative features of cirrhosis including nodular liver contour, splenomegaly, enlarged main portal vein, or varices. Subjects for whom the diagnosis of cirrhosis remained uncertain were reviewed by two hepatologists until consensus was reached.

Response to treatment was determined by ALT levels. For patients on AIH therapy for six or more months, complete response was defined as AST < 30 U/L and ALT ≤ 19 U/L or ≤ 30 U/L for women or men, respectively. Partial and non-response were defined as ALT ≤ 60 U/L and ALT >60 U/L, respectively.

### Extraction and sequencing library preparation

**Fig 1:**
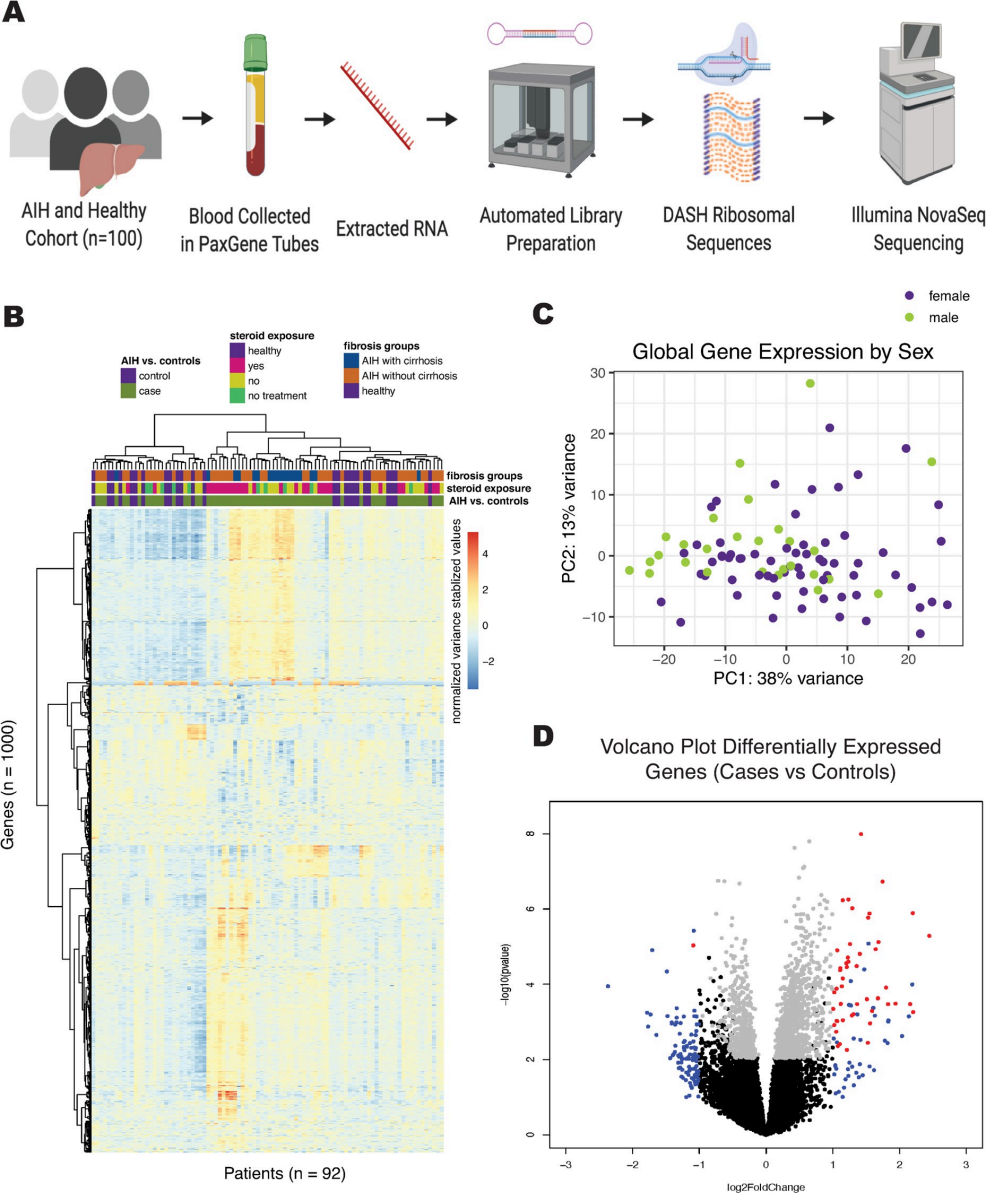
A. Schematic of sample processing for library preparation of whole blood for AIH cohort. B. Heat map displaying gene counts (variance stabilizing transformation, see DESeq2) of the top 1000 genes with highest variance amongst samples. Samples are clustered on the x-axis and protein coding genes are clustered on the y-axis using a Ward D2 method. Relevant metadata included patient status, steroid exposure, and fibrosis groups to classify samples. C. PCA plot of variance stabilizing transformed gene counts colored by sex of patient sample. D. Volcano plot of differential gene expression in DESeq2, showing genes with P < 0.05 and log fold change > 1 in red, and genes with only log fold change > 1 in blue, and genes with only P < 0.05 in grey.

Blood from GRACE participants had been stored in PAXgene tubes and frozen at -80°. After thawing, RNA was extracted from whole blood using the Qiagen PAXgene Blood RNA System [10]. Quantification was performed using Qubit RNA HS Assay (Thermo Fisher Scientific) and analysis of RNA integrity was performed using Fragment Analyzer DNF-472 High Sensitivity RNA (Advanced Analytical). RNA was normalized to 2ng/uL or less in 5uL based on Qubit values. External RNA Controls Consortium (ERCC) controls (Thermo Fisher Scientific) were spiked into the samples by adding 1μL at 25pg/μL as quantified by Qubit. Reverse transcription and library preparation were performed using New England Biolab’s NEBNext Ultra II RNA Library Prep (E7770), with an RNA fragmentation time of 8 minutes and 18 cycles of indexing PCR using custom TruSeq indexing primers. Samples were prepared in two batches using the Agilent Bravo Automated Liquid Handling Platform. A water control was included in each batch. For each ten of the RNA samples, a technical replicate was included. Prior to deep sequencing on a NovaSeq (Illumina), relative library concentrations of each sample were determined by pooling 1μL of each and sequencing on a MiSeq (Illumina) with an average of 360,000 read pairs/sample (Fig 1A).

### Depletion of Abundant Sequences by Hybridization (DASH)

DASH is a CRISPR-Cas9 technology which allows targeted depletion of uninformative sequences from a pooled library using a custom gRNA set [11]. Initial MiSeq sequencing enabled prediction of the effect of depletion of human nuclear and mitochondrial ribosomal RNA (rRNA) using DASHit. It was found that DASH targeting human rRNA would free up 90.7% of sequencing space on average. Samples were pooled to achieve equimolar non-rRNA concentration. Analysis of library concentration and size was performed using the Tapestation High Sensitivity D5000 (Agilent Technologies). Pools were normalized to 100ng in 23μL using Qubit HS DNA (Thermo Fisher Scientific) and 2 additional amplification cycles were performed using KAPA’s Real-time Library Amplification kit (KK2702) to reduce effects of prior overamplification. Pools were then normalized to 2.8nM using Qubit HS DNA (Thermo Fisher Scientific).

DASH was performed on the pools using a gRNA set targeting human rRNA [11]. Cas9 was purified from E. coli as described, and gRNA were transcribed from templates ordered from Integrated DNA Technologies using T7 polymerase as described. Cas9-gRNA complex was incubated at 37°C for 2 hours with pooled samples and reactions were quenched with Proteinase K. Samples were further amplified using KAPA’s Real-time Library Amplification (KK2702).

### Sequencing

Libraries were sequenced on Illumina NovaSeq 6000 using 150bp paired-end reads. Samples received an average of 42 million read pairs/sample.

### Gene counts

Sequencing data was quality filtered using PriceSeqFilter [12], with parameters specifying that 90% of nucleotides in a read pair must be called with the flag “-rnf 90” and 85% of nucleotides in a read pair must have a 98% probability or higher of being correct with the flag “-rqf 85 0.98”. TruSeq adaptors were trimmed using cutadapt, with a minimum length of read remaining set to 36 [13]. Data was then run through IDSeq (PIPELINE v2.8, NT/NR: 2018-04-01), a pathogen-detection pipeline developed by the Chan-Zuckerberg Initiative [14]. Gene counts were obtained from the initial step in the IDSeq pipeline, Spliced Transcripts Alignment to a Reference (STAR) [15] alignment to human reference genome 38 (hg38). All hemoglobin genes and genes which did not code for proteins were removed based on annotations in BioMart v2.34.2 [16]. In addition, a filter for genes with low counts was applied to remove genes that had fewer than 10 reads in 20% or more of the samples.

### Differential gene expression

Genes which had less than 10 total reads in fewer than 20% of the samples were filtered out of the gene counts. Differential gene expression was performed by inputting unnormalized gene counts into the DESeq function of Bioconductor package DESeq2 (v1.18.1), using default settings without a normalization matrix. The DESeq function estimates size factors (normalization for library size included) and dispersion factors for negative binomial distributed data, then runs a Wald test to determine significance of coefficients of the negative binomial GLM of the data. Design matrix modelled sex, age, library prep batch and our variable of interest (ex: ~ library batch + sex + age + AIH status. Variables of interest used for each analysis is indicated in discussion section and includes AIH vs healthy controls, steroid exposure (healthy vs treatment naive vs steroid-containing treatment vs steroid-sparing treatment) and fibrosis groups (AIH with cirrhosis vs AIH without cirrhosis vs healthy). Differential gene lists were generated by pairwise comparison of the groups (i.e., AIH vs healthy controls or treatment naive vs healthy controls) with the results function of DESeq2, with an adjusted p-value cut-off of 0.05.

### Pathway enrichment analysis

Differential gene expression results were imported into Ingenuity Pathway Analysis software (Qiagen, Redwood City, CA, USA). Genes were considered for entry into the analysis if they had an absolute log fold change greater than 0.6 between the two groups of interest (cases vs. controls, or treatment-naïve AIH vs. controls), but no adjusted p-value cutoff was required.

### Defining an AIH-specific gene signature using random forest

The random forest method (a classifier that combines many single decision trees) was used to calculate the importance of each gene for defining AIH vs control [17]. The varSelRF R package uses the out-of-bag error as the minimization criterion and carries out variable elimination with random forests by successively eliminating the least important variables (with importance as returned from the random forest analysis) [18]. The algorithm iteratively fits random forests, at each iteration building a new forest after discarding genes with the smallest variable importance; the selected set of genes is the one that yields the smallest out-of-bag error rate. This leads to the selection of small sets of non-redundant variables. To examine the discriminatory power of this metric, we varied the standard deviation cutoff by using multiplication factors from 1 to 2 in steps of 0.25.

### Weighted gene correlation network analysis

A total of 8,173 genes (identified as described in gene counts, above), as well as clinical metadata variables, were used to construct a module-trait graph to identify relationships between clusters of genes and clinical variables. This analysis was performed using the Weighted Gene Correlation Network Analysis algorithm in R as previously reported [19]. Briefly, genes were analyzed using the Pearson’s correlation test, and a matrix of similarity was constructed with a soft power of β = 6. The adjacency matrix of gene expression data was clustered using topological overlap matrix analysis. A dendrogram was then constructed using average linkage hierarchical clustering and the dynamic tree cut algorithm was applied to the dendrogram for module identification. To identify hub genes, we used a gene significance cutoff of 0.5 and a measure of centrality within the module, module membership, at a cutoff of 0.7. To assess the strength of the correlation between genes of interest and individual modules, gene significance for the genes in the module were compared to module membership using univariate regression, and correlation p-values were generated.

### Cibersort

We performed deconvolution of bulk RNASeq data (filtered normalized protein coding gene counts as described above) using the CIBERSORT algorithm, which we ran through the web interface using default parameters, as previously reported [20].

## Results

### Clinical cohort

75 AIH patients in the GRACE Cohort provided whole blood samples, from which RNA was isolated. After excluding patients with concomitant liver disease (n = 2), those who had undergone liver transplantation (n = 4), and those whose sequencing were extreme outliers determined by hierarchal clustering and rRNA content (n = 2), there were 67 AIH patients and 25 healthy volunteers with whole blood RNA-Seq data (Table 1). The median age was 54 years for AIH patients, and 50 years for healthy volunteers. The AIH cohort was predominantly female (79%), whereas healthy volunteers were 48% female. Both groups were predominantly white. Median ALT was 31 U/L in the AIH patients and 19 U/L in the healthy volunteers. Among AIH subjects, 56 (84%) had Type 1 AIH, 1 (1%) had Type 2 AIH, 2 (3%) had drug-induced AIH, and 8 (12%) had AIH of unknown type. Autoantibody results were available as follows: 32 of 51 subjects were ANA positive; 41 of 50 subjects were positive for anti-actin antibody; only 1 of 19 subjects was positive for anti-LKM-1 antibody. At the time of blood collection, 27% of the AIH subjects had cirrhosis and 7% were treatment-naïve. 10 AIH patients were off therapy at the time of blood biospecimen collection (5 were treatment-naïve, 3 were in remission and had been withdrawn from therapy, and 2 had been previously treated but were off therapy at the time of sample collection). Of subjects on AIH therapy at the time of sample collection, 28 were on a regimen that included corticosteroids: 21 with and 7 without maintenance medications. 29 of the AIH patients on therapy at the time of sample collection were on a steroid-free regimen consisting only of maintenance medications such as azathioprine, mycophenolate, or tacrolimus. Of those who had been on therapy for ≥6 months at the time of blood collection, 27% were in complete remission.

**Table 1:** Clinical cohort of AIH patients and healthy controls.

Characteristics	AIH (n = 67)	Healthy (n = 25)
Age, y, median (range)	54 [20–79]	50 [24–70]
*Sex*, *n (%)*		
Men	14 (21%)	13 (52%)
Women	53 (79%)	12 (48%)
*Race/ethnicity*		
White	60 (90%)	25 (100%)
People of Color	7 (10%)	0 (0%)
ALT, U/L, median (range)	31 [4–936]	19 [10–34]
IgG, mg/dL, median (range)	1540 [201–3890]	
Cirrhosis	18 (27%)	
Decompensated	12 (18%)	
*Treatment Status*		
Treatment-naive	5 (7%)	
Previously-treated	5 (7%)	
On therapy ≥6 months		
Complete Remission	18 (27%)	
Partial Response	18 (27%)	
Non-responder	6 (9%)	
Required change in treatment due to prior non-response	12 (18%)	
*Treatment Type*		
Steroid-containing regimen	28 (42%)	
Steroid-free regimen	29 (43%)	
Off treatment	10 (15%)	

### Gene expression profiles can differentiate AIH patients from healthy controls

To identify peripheral blood gene expression profiles unique to AIH, we compared AIH subjects to healthy controls (Fig 1B). Sequencing yielded an average of 42 million read-pairs per sample. Full data set available at https://github.com/czbiohub/AIH-Project. Unsupervised clustering of the 1000 most variably expressed genes in the dataset did not entirely separate samples based on diagnosis, treatment, or fibrosis stage. Unsupervised clustering analysis on all gene counts did not reveal grouping of samples by demographic factors such as age, sex, and race, nor by RNA preparation batch (Fig 1C, S2 Fig).

Analysis of global trends in gene expression identified 249 differentially-expressed genes (Fig 1D); the top 20 genes with greatest fold-change are listed in S1 Table. Random forest modeling with recursive variable elimination indicated that 9 or fewer genes can reliably differentiate AIH patients from healthy controls (Table 2).

**Table 2:** Random forest results with increasing standard deviation multiplicative factor separating AIH patients and healthy controls.

Standard Deviation Allowed	Number of Genes Identified	Genes Identified by Random Forest (% of seeds gene appeared)
1	9	ARHGAP4 (30%), COL5A3 (50%), EZH1 (10%), FLYWCH1 (30%), GIGYF1 (100%), MAPK8IP3 (20%), MEGF6 (20%), PTOV1 (90%), RHOT2 (10%)
1.25	7	ARHGAP4 (50%), COL5A3 (60%), FLYWCH1 (70%), GIGYF1 (90%), MAPK8IP3 (10%), MEGF6 (10%), PTOV1 (100%)
1.5	2	GIGYF1 (100%), PTOV1 (100%)
1.75	5	COL5A3 (10%), FLYWCH1 (30%), GIGYF1 (100%), PTOV1 (80%), SLC4A10 (10%)
2	3	FLYWCH1 (20%), GIGYF1 (90%), PTOV1 (90%)

### Interferons are the predominant upstream regulators of gene induction in AIH

**Fig 2:**
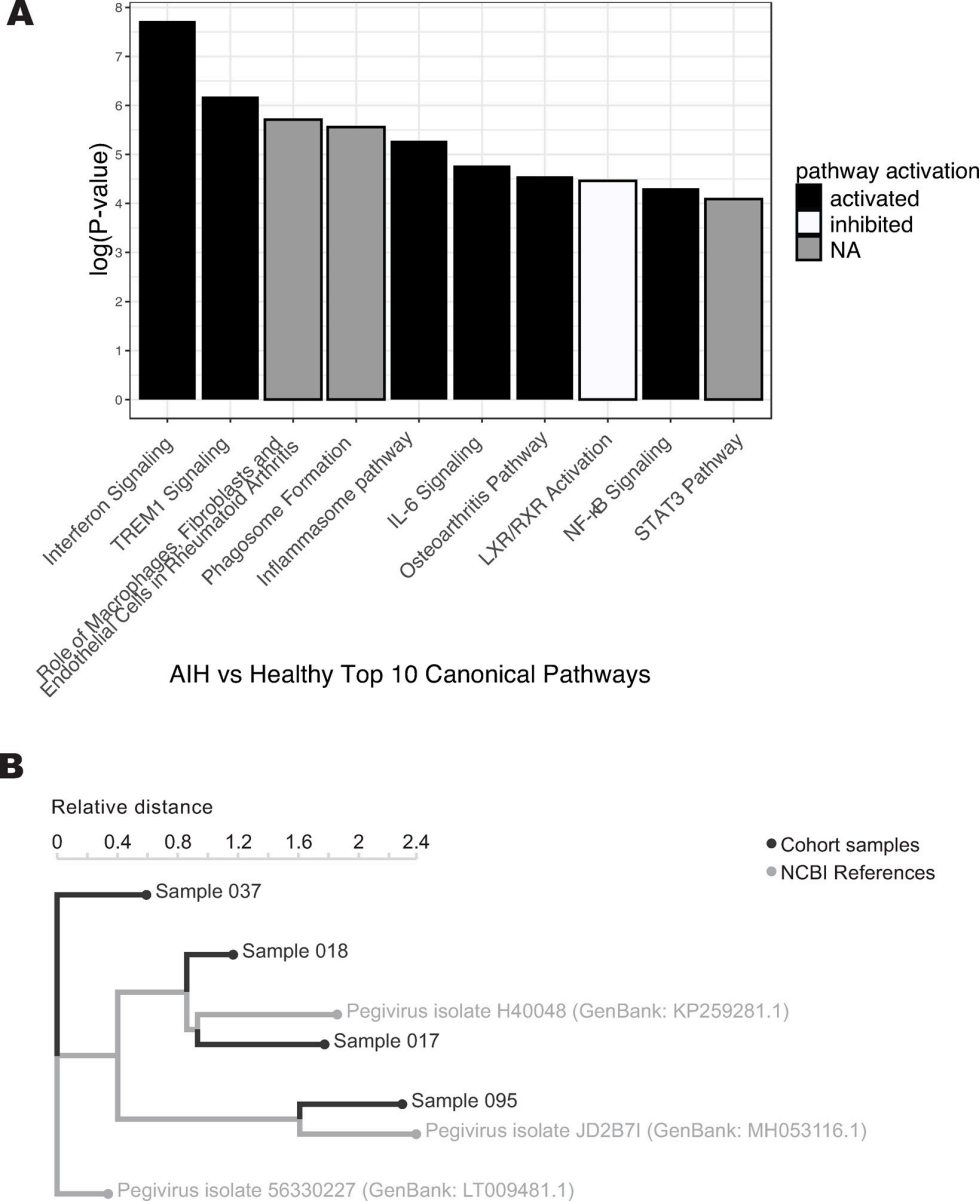
A. Top ten most significant canonical pathways related to differential gene expression of AIH patients compared to healthy controls. B. kmer-based phylogenetic analysis performed with the IDSeq platform shows relationships between four assembled pegivirus genomes from the GRACE cohort and their closest related publicly available pegivirus NCBI reference genomes, with patient metadata annotated.

We performed pathway analysis in order to identify patterns across genes that were enriched in AIH patients relative to healthy controls. Across all AIH subjects, interferon signaling was the canonical pathway most likely to be differentially regulated (by p-value), activated in AIH subjects relative to healthy controls (p < 0.0001, Fig 2A). Analysis further identified several additional pathways of interest, for example, myeloid signaling, as indicated by activation of triggering receptor expressed on myeloid cells 1 (TREM1) and inflammasome pathways, but inhibition of liver X receptor/retinoid X receptor (LXR/RXR) activation (Fig 2A).

To eliminate the impact of immunosuppression on the interferon signal, we next compared treatment-naïve subjects (n = 5) to healthy controls (n = 25). Interferon signaling remained one of the top 10 activated canonical pathways (S3 Fig). However, for the majority of pathways, the directionality (activation vs. inhibition) could not be determined. To identify whether a cascade of transcriptional regulation could explain the observed gene expression changes, we performed upstream regulator analysis in Ingenuity Pathway Analysis which revealed 515 significant pathways (S2 Table). The top 10 activated upstream regulators, sorted by p value, is shown in Table 3A. Of these 10 genes, five were interferon genes (IFNA2, IFNG, IFNL1, IRF7 (a master regulator of type I IFN signaling [21, 22]), and IFN downstream response gene 1 [23], confirming the central role of interferon signaling in autoimmune hepatitis. This is consistent with signatures obtained across other autoimmune diseases such as systemic lupus erythematosus [24–26] and with the known association between treatment with IFN-a and development of autoimmune hepatitis [27, 28].

**Table 3:** A. Top 10 activated upstream regulators identified by pathway analysis for treatment-naïve patients compared to healthy controls. **B.** Top 10 inhibited upstream regulators identified by pathway analysis for treatment-naïve patients compared to healthy volunteers.

Upstream Regulator	Predicted Activation State	Activation z-score	p-value of overlap	Target molecules in dataset
IRF7	Activated	3.898	1.63E-12	CMPK2,GBP1,IFI44,IFI44L,IFI6,IFIT1,IFIT3,IFITM3,ISG15,LILRA5,OAS3,OASL,RIPK2,RSAD2,S100A8,TNFSF13B
IFNG	Activated	3.828	9.57E-09	AIF1,B2M,CMPK2,CYP27A1,FCGR1B,FTL,GBP1,GNAS,HLA-DRA,HLA-DRB5,HSPB1,IFI44,IFI44L,IFI6,IFIT1,IFIT3,IFIT5,IFITM3,ISG15,KLF1,LGALS3,MYH9,OAS3,OASL,PCTP,PHB2,PRDX2,PRPF8,RIPK2,RSAD2,S100A8,S100A9,TFRC,TGFBR3,TNFSF13B
MYCN	Activated	3.592	4.33E-15	ABCB10,B2M,CLU,LGALS1,MYH9,RPL23,RPL27,RPL35,RPL41,RPL6,RPL7,RPL9,RPLP0,RPS13,RPS25,RPS27,RPS3A,RPS5,RPS6,RPS8,RPS9,SPARC
IFNA2	Activated	3.57	2.32E-09	ANXA1,B2M,CMPK2,GBP1,GNAS,IFI44,IFI44L,IFI6,IFIT1,IFIT3,IFIT5,IFITM3,ISG15,OAS3,RSAD2
IFNL1	Activated	3.554	3.64E-13	CMPK2,GBP1,IFI44,IFI44L,IFI6,IFIT1,IFIT3,IFIT5,IFITM3,ISG15,OAS3,OASL,RSAD2
IRF1	Activated	3.37	6.03E-08	B2M,CMPK2,IFI44L,IFI6,IFIT1,IFIT3,IFIT5,IFITM3,ISG15,OASL,RSAD2,TNFSF13B
PAF1	Activated	2.646	8.13E-09	HIST1H2BD,IFI44,IFI44L,IFIT3,IFITM3,ISG15,OAS3,OASL
ribavirin	Activated	2.63	0.000000062	IFI44L,IFI6,IFIT3,ISG15,OASL,RNF125,RSAD2
PRL	Activated	2.515	2.27E-08	B2M,CLU,CMPK2,EPSTI1,IFI44,IFI44L,IFI6,IFIT1,IFIT3,IFIT5,ISG15,OAS3,RSAD2,SPARC,TNFSF13B
TCR	Activated	2.041	1.02E-11	BSG,IFI44,IFI44L,IFI6,IFIT1,IFIT3,ISG15,OASL,PASK,PHB2,PRDX6,RPL6,RPL7,RPL9,RPLP0,RPS13,RPS3A,RSAD2,SLC1A5,TNFSF13B,UBA52
sirolimus	Inhibited	-3.798	7.4E-13	B2M,CDA,COX6B1,CREG1,FUS,ISG15,LGALS3,RPL23,RPL27,RPL35,RPL41,RPLP0,RPS13,RPS18,RPS21,RPS27,RPS27A,RPS29,RPS3A,RPS5,RPS6,RPS8,RPS9,SLC1A5,TUBB2A,UBA52
IL1RN	Inhibited	-3.317	1.45E-08	GBP1,IFI44,IFI44L,IFI6,IFIT3,IFIT5,OAS3,OASL,RIPK2,RSAD2,S100A9
ST1926	Inhibited	-3.317	0.000000153	CHCHD2,COX7A2L,GNAS,LGALS1,PSMF1,RPL41,RPL6,RPS27,RPS27A,TPT1,UBA52
RICTOR	Inhibited	-3.303	1.89E-13	BSG,COX6B1,COX7A2L,ISG15,PSMF1,RPL23,RPL41,RPL6,RPL7,RPL9,RPLP0,RPS13,RPS18,RPS21,RPS27A,RPS29,RPS5,RPS6,RPS8,RPS9
MAPK1	Inhibited	-3.207	0.0000023	GBP1,IFI44,IFI6,IFIT1,IFIT3,IFIT5,IFITM3,ISG15,ITGA2B,LGALS1,LGALS3,OAS3,OASL,RGS2
ACKR2	Inhibited	-2.449	0.00000168	IFI44,IFIT3,ISG15,OAS3,OASL,RSAD2
filgrastim	Inhibited	-2.433	4.7E-12	ANXA1,B2M,CLC,CLU,CYP27A1,CYSTM1,EPSTI1,GBP1,HLA-DRA,HLA-DRB5,IFI44,IFI6,IFIT1,IFIT3,IFIT5,ISG15,LGALS3,LILRA5,OAS3,OASL,PRDX2,RSAD2,SPECC1
ZNF106	Inhibited	-2.333	1.09E-10	COX7A2L,HSPB1,IFITM3,LGALS1,LGALS3,LYZ,PRDX2,S100A8,TMEM106B
SP110	Inhibited	-2.309	3.22E-09	ATF2,CLU,ETS1,FUS,IFI6,IFIT1,IFIT3,IFITM3,MYH9,OAS3,RIPK2,TGFBR3
TRIM24	Inhibited	-2.121	0.00000232	CMPK2,EPSTI1,IFI44,IFIT3,ISG15,LGALS3,OASL,PLEC

Analysis of the top 10 inhibited upstream regulators (Table 3B) showed that four were known therapies for AIH or other disease states: sirolimus, ST1926 (a synthetic retinoic acid), IL1RN (anakinra), and filgrastim [29–32]. Given that inhibition of genes downstream of these master regulators are suppressed in AIH patients relative to controls, these upstream regulatory networks may represent prime targets with therapeutic potential in AIH.

### Pathogen detection platform reveals infection with pegivirus in a handful of samples, but cannot fully explain the activation of interferon signaling in AIH compared to healthy controls

Of the 75 AIH subjects and 25 healthy controls, IDSeq identified pegivirus (formerly known as hepatitis G) sequences in whole blood specimens from seven patients: five AIH subjects, one AIH and hepatitis C subject, and one healthy control. In four, pegivirus read depth was high enough to place on a kmer-based phylogenetic tree along with their closest NCBI reference sequences using the IDSeq platform (Fig 2B). Variation in viral strains precludes the possibility that these infections represent a cluster among the GRACE cohort population. The presence of pegivirus in whole blood is compelling but is insufficient to explain the extent of interferon induction we observed, given that interferon signaling was activated in many samples beyond those with pegivirus viremia.

### Gene expression correlates with fibrosis stage in AIH

**Fig 3:**
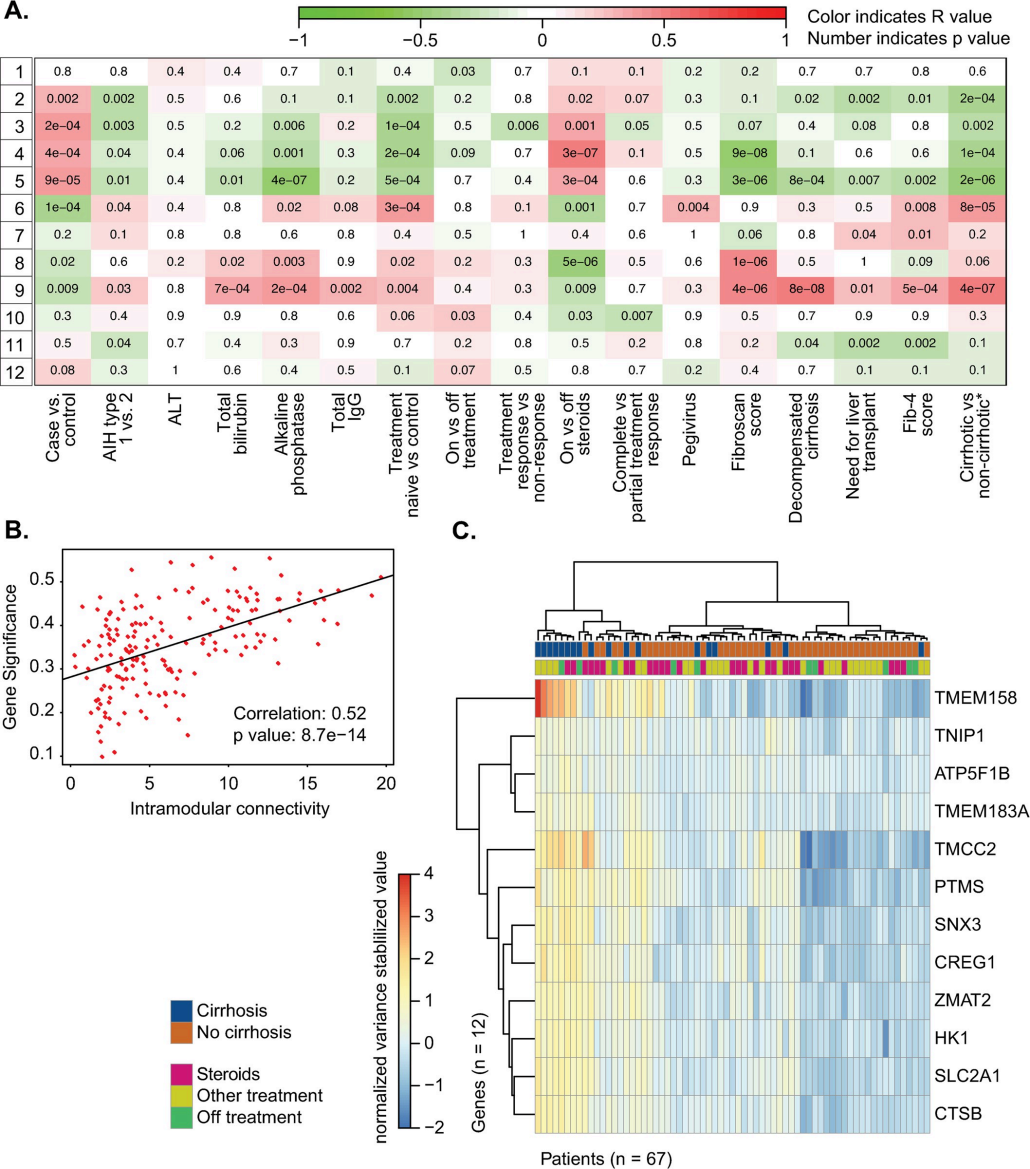
A. WGCNA module trait graph, with patient metadata on the x-axis and generated gene modules on the y-axis. B. Interconnectivity plot for gene module 9 identified by WGCNA. C. Heatmap displaying gene counts (variance stabilizing transformation, see DESeq2) of the top 12 hub genes identified from interconnectivity metrics as described in Methods. Samples are clustered on the x-axis using a Ward D2 method and color annotated with cirrhosis status and treatment at sample collection.

Weighted Gene Correlation Network Analysis was used to create clusters of highly correlated genes, also known as gene modules. These gene modules were then related to clinical variables of interest in order to identify relationships between clinical phenotypes and gene expression (Fig 3A). Using this approach, we found 12 gene modules. We observed that gene module 9 correlated with not only the greatest number of clinical variables of interest but also markers of poor AIH outcomes in AIH. Specifically, gene module 9 was associated with decompensated cirrhosis (p < 0.001), the need for liver transplantation (p = 0.01), two surrogates of liver cirrhosis, Fib-4 (p < 0.001) and transient elastography scores (p < 0.001), and a composite variable of cirrhosis, as determined using the algorithm in S1 Fig (p < 0.001). Conversely, module 5 was significantly inversely correlated with clinical variables related to advanced fibrosis (fibroscan score, FIB-4 score, cirrhosis, decompensation, and need for transplant). This correlation between a module of genes and the presence of liver cirrhosis is striking, as cirrhosis on index liver biopsy is known to portend a poor prognosis in patients with AIH [33].

The correlation between liver cirrhosis and the genes in module 9 is measured by gene significance; in Fig 3B this correlation value is plotted against a measure of how connected that gene is to other genes within module 9 to identify the most central genes within the module. We next identified key hub genes within module 9 that separate AIH subjects with and without cirrhosis as previously reported [34] and described in methods. This yielded a list of 12 top hub genes (Fig 3 and S3 Table). Plotting these 12 genes in a heatmap using hierarchical clustering resulted in partial separation of the cirrhotic and non-cirrhotic AIH subjects (Fig 3C).

### Complete response to treatment is associated with higher blood CD8 T cell counts

**Fig 4:**
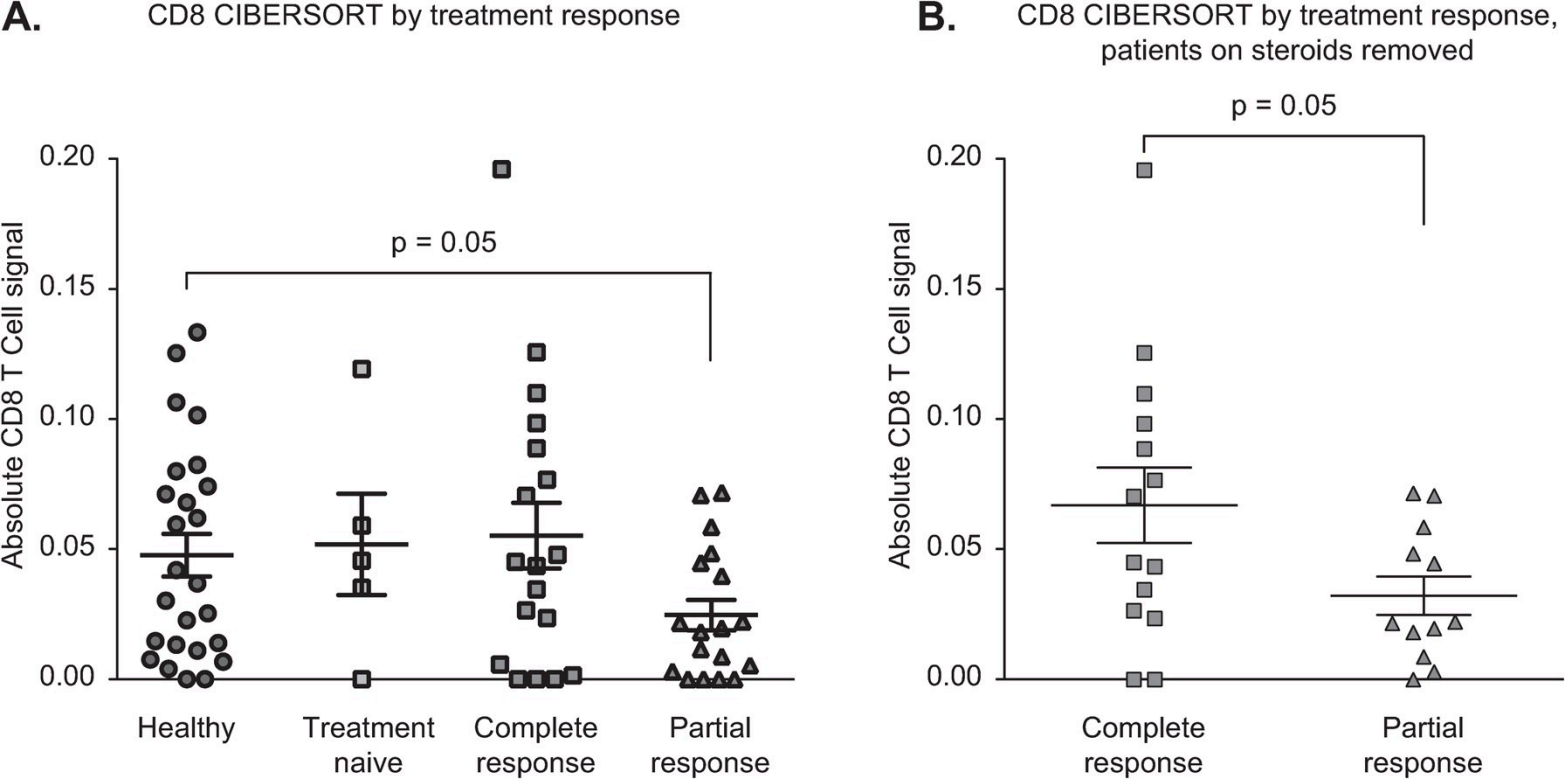
A. CIBERSORT data deconvoluting absolute CD8+ T-cell counts from bulk RNA-seq, with significant differences in counts between healthy patients and those with a partial response to treatment and between those with a complete compared to a partial response to treatment. B. CIBERSORT data deconvoluting absolute CD8+ T-cell counts from bulk RNA-seq, removing steroid patients and showing a significant difference between those with a complete compared to a partial response to treatment.

Application of bulk RNA-Seq analysis to whole blood allows for assessment of both viral and cellular RNA, making it possible to assess immunologic response, in our case interferon signaling, while simultaneously searching for possible viral triggers. In parallel, we sought to determine the contribution of individual immunologic populations on AIH pathobiology through deconvolution of bulk RNASeq data using the CIBERSORT algorithm, which we ran through the web interface using default parameters [20]. We compared the leukocyte profiles in AIH subjects and healthy controls to determine whether subsets varied according to response to therapy. Specifically, while incomplete response to immunosuppression has been linked to poor long-term outcomes among AIH patients [35], specific clinical characteristics or a mechanistic understanding of *a priori* determinants of response remain limited [33]. We compared gene expression of healthy controls (n = 25) to treatment-naive AIH subjects (n = 5) and AIH subjects with either complete (n = 18) or partial (n = 18) response. AIH subjects with partial response to therapy (n = 18) had a lower CD8 T cell signature in the periphery compared to healthy controls (p = 0.05) and compared to complete responders (p = 0.04) (Fig 4A). After excluding patients on corticosteroids from the analysis, partial responders still had a lower CD8 T cell signature than complete responders (p = 0.05, Fig 4B).

## Discussion

A major barrier to addressing disparities and improving the management of autoimmune hepatitis (AIH) is the disease’s obscure etiopathogenesis. In an effort to promote precision medicine in a field where prednisone has been a primary therapy for several decades, we applied state-of-the art RNA sequencing to whole blood specimens from a well-described cohort of AIH patients and healthy volunteers.

We found 249 genes that were significantly differentially expressed in the whole blood of AIH patients compared to healthy controls. This relatively small number of differentially expressed genes could be related to the fact that most blood samples were drawn from AIH patients after the initiation of therapy, heterogeneity among cases and/or, or perhaps the need to focus on certain cell types within whole blood. We performed PCA on covariance of the genes and didn’t find obvious clusters when looking at cases vs. controls. While sex was not clearly delineated in PC1 or PC2 of our principal component analysis, this does not rule out the possibility that sex could be a minor confounder. However, we did see hierarchical clustering of genes when we focused on the 1000 most highly expressed genes in the dataset (see Fig 1B). The strongest signal of genes differentially expressed in AIH compared to healthy controls was that of interferon activation. Interferons are pro-inflammatory cytokines and part of the innate immune system, traditionally viewed as the body’s first line of defense against pathogens. Interferons play an important role in the liver infected with hepatitis B or C virus and indeed have been used as an antiviral therapy. In fact, autoimmune hepatitis is a well-recognized complication and relative contraindication to interferon-based antiviral therapy [28]. A prior study by Grant et al. showed that CD4+ cells cultured *in vitro* from the peripheral blood of treatment-naïve AIH patients produce interferon-gamma for an extended period of time, beyond that seen in healthy samples [36]. In addition, another study of treatment-naïve AIH patients found that serum interferon-gamma-inducible protein-10 (IP-10) levels were significantly correlated with histologic inflammation [37]. AIH was historically coined “lupoid hepatitis,” and recent work has highlighted interferon as a therapeutic target for the drug anifrolumab in systemic lupus erythematosus patients [38].

In light of the robust interferon signal, it was logical to query for the presence of a virus. One significant advantage of sequencing whole blood was the ability to answer this question, and pegivirus sequences were identified in the blood of six patients with AIH. Pegivirus has no confirmed pathogenic effect [39]. It has both liver and immune cell tropism [40], and studies have shown that infection with HBV or HCV increase the risk of pegivirus infection [41]. Pegivirus is more prevalent in patients with liver transplant than partial hepatectomy patients, but pegivirus is not associated with any changes in clinical outcomes [42]. Notably, a recent systematic review and metanalysis of studies of pegivirus RNA prevalence in healthy blood donors in 2019 revealed a global prevalence of 3.1% and a North America prevalence of 1.7% [41]. Prevalence varies significantly by geographic region, with some studies reporting prevalence of over 20% [41]. Anti-pegivirus antibody prevalence is higher, suggesting that the virus is frequently cleared [40, 41]. Given that there is no molecular evidence yet pointing towards pegivirus as a causative agent in AIH or any other disease, we note that increased susceptibility to viral infection in patients treated with immunosuppressive therapy may account for the higher incidence in this AIH cohort [41]. However, the activation of interferon signaling in the AIH group as a whole raises the possibility that viral infection could play a role in disease etiology, possibly as a trigger for molecular mimicry, something which warrants further study. Interestingly, pegivirus is structurally very similar to hepatitis C virus. There is a small body of literature linking HCV to AIH, with recent data suggesting that clearance of HCV can lead to resolution of AIH activity in some patients [43]. Furthermore, TP4502D6 (CYP2D6) is an autoepitope recognized by anti-LKM in patients with Type 2 AIH and patients with HCV [44].

In order to focus on disease pathophysiology, AIH patients who submitted blood samples prior to treatment initiation (treatment-naïve patients) were studied in a separate analysis. Among the multiple findings, interferon signaling was an activated canonical pathway. Regarding inhibition, genes that would be found downstream of the drug sirolimus were inhibited in AIH patients. The most significantly inhibited upstream regulators of gene expression in treatment-naïve patients compared to healthy included several druggable targets. RPTOR Independent Companion Of MTOR Complex 2 (RICTOR) was determined by pathway analysis to be a significantly inhibited upstream regulator of gene expression in treatment-naïve AIH patients, with multiple target molecules supporting this in the dataset. Sirolimus is a macrolide drug that inhibits mTOR, a protein that controls the proliferation and survival of activated lymphocytes. Indeed, mTOR signaling was one of the most significant pathways in our focused analysis of treatment-naïve patients vs. healthy controls (S3 Fig). The Grant et al. study cited above also found that sirolimus increases the *in vitro* responsiveness of lymphocytes to regulatory T cells [36]. Sirolimus has been studied as a salvage therapy for AIH in reports of very small numbers of patients, with mixed results [29]. However, like many second-line therapies for AIH, it has never been studied in a randomized controlled trial. Our results suggest a biologic basis for the use of sirolimus in AIH and that it merits further study as a therapeutic agent. Filgrastim, or G-CSF, was also predicted to be an inhibited upstream regulator of gene expression in the AIH samples. While never studied in AIH, G-CSF has been studied as a treatment for severe acute alcohol-associated hepatitis with some success [32, 45]. In alcohol-associated hepatitis, G-CSF is believed to mobilize CD34+ stem cells from the bone marrow to the liver, where they promote regeneration in the setting of severe inflammation. Given this mechanism, it is plausible that G-CSF may have a role in the treatment of AIH.

We also used a random forest algorithm to determine a small set of genes that distinguish AIH patients from healthy volunteers. The genes predicted by the model do not specifically indicate AIH disease biology but can potentially serve as biomarkers in a diagnostic test to flag a patient for further analysis of AIH status. The diagnosis of autoimmune hepatitis can be challenging, often requiring an invasive liver biopsy and the calculation of diagnostic scores based on multiple clinical factors [6]. Given the current lack of a single accurate diagnostic test for AIH, a biomarker in the peripheral blood would be immensely helpful to the clinician. However, we recognize that many of these genes appear in less than half of seeds and these may not be stable predictors when applied to validation datasets. We acknowledge that our predictive power is greatly limited by the small size of this pilot cohort, and emphasize that this algorithm does not have clinical significance without further testing and validation.

In multiple studies, cirrhosis has been linked with poor outcomes such as death and liver transplantation in AIH [46–48]. 30–40% of AIH patients have cirrhosis on index liver biopsy. While some patients have obvious radiographic evidence of cirrhosis, there are many patients with early, compensated cirrhosis (i.e. without clinically evident complications), for whom fibrosis stage is only apparent on a liver biopsy. Noninvasive methods of fibrosis assessment such as transient elastography and magnetic resonance elastography, while validated in AIH, are not available or accessible for many AIH patients. Therefore, a noninvasive blood signature of cirrhosis in AIH could be very valuable in risk stratifying AIH patients. We used gene correlation network analysis to determine a gene expression signature for advanced disease (stage 3–4 fibrosis) from this dataset. If validated in a different cohort, this 12-gene signature could provide insight into more severe cases of AIH and serve as a prognostic marker. While a blood test for cirrhosis in AIH would be incredibly helpful for predicting prognosis, one caveat is that the gene expression signature identified in this study was associated with cirrhosis in a heterogenous group of AIH patients. Some patients had already decompensated with events such as variceal hemorrhage. Others had been diagnosed several years prior to blood sample collection. In contrast, prior studies linking cirrhosis with worse survival in AIH used biopsy results at the time of diagnosis. Therefore this gene expression signature needs to be validated in a prospective fashion but retains value as a potentially powerful tool to identify AIH patients at higher risk for poor outcomes.

Finally, dividing the AIH cohort by response to treatment and deconvoluting the gene expression data by cell type pointed to a role for CD8+ T cells. Patients with a partial response to therapy displayed a lower CD8+ T cell expression signal. CD8+ T lymphocytes are cytotoxic and induce apoptosis of damaged cells in response to antigen presentation on MHC Class I molecules, and they are a major cell type in areas of interface hepatitis [49]. In Type II AIH, the degree of CD8+ T cell response correlates with disease activity [50]. *In vitro* studies have shown that immunosuppressive therapy alters the ability of regulatory T cells to modulate CD8+ T cell activity [51]. However, these results are limited by the fact that the effect of AIH on CD8 cell populations is difficult to truly separate from the effect of AIH therapies, i.e. various forms of immunosuppression. Further study is required to better understand the relative contribution of immunosuppression and underlying AIH disease activity on CD8 biology. It has been shown that interferon alpha/beta induction inhibits egress of lymphocytes from lymphoid organs [52]. While complete responders may have a CD8 response that is “restored” or closer in character to healthy controls given their treatment response, partial responders may still have active AIH disease activity, i.e. a CD8 signature more similar to active AIH. Our results suggest that those with incomplete response to therapy have fewer or less functional circulating CD8 T cells. This also raises the possibility that in partial responders, CD8 T cells may have left the periphery and entered the liver, where these cells could lead to further inflammation and tissue destruction. These findings may provide insight into why some patients enjoy a complete response to therapy while others have ongoing disease activity or even experience hepatic decompensation.

A weakness of this pilot study is its relatively small sample size, which did not allow us to divide the patients into training and validation sets. The small number of AIH patients who provided blood samples prior to treatment initiation limited our power to detect disease signals independent of medication effects. With the small number of treatment-naive patients, our analysis of pathways activated or inhibited in this group could not use a p-value cutoff for genes considered in the analysis and was thus exploratory only. For the analysis of AIH cases vs. healthy controls, the pathway results were similar with or without a p-value cutoff for included genes. In addition, this relatively recently formed patient cohort had limited follow-up data, so we were not able to look at gene expression as a predictor of subsequent clinical outcomes. We did not have liver biopsy data on all patients but developed an algorithm for determining if patients had cirrhosis, which raises the possibility of bias in our analysis. Moreover, this pilot study did not include patients with other types of liver diseases aside from AIH. However, further efforts to expand longitudinal cohorts that include AIH patients, clinical data, and blood and liver biospecimens are currently underway, and classical immunophenotyping assays are planned.

Nevertheless, this study represents a unique contribution to the AIH field both by shedding light on molecular mechanisms and opening possibilities of better management. Cutting-edge genomic technology was applied to a large cohort of AIH patients with detailed clinical data. Whole blood samples were utilized, allowing for signals from cells not typically included in immunologic studies of AIH, such as platelets and neutrophils. The unbiased and comprehensive approach of RNA sequencing yielded results that corroborated prior in vitro studies of the role of interferon and CD8+ T cells in AIH. We also uncovered a number of AIH samples with viral sequences, an unexpected finding that raises the possibility of molecular mimicry after viral infection as a mechanism in AIH. We were able to develop two gene signatures—one for disease and one for cirrhosis—which will need to be validated in future studies but serve as proof-of-principle that new biomarkers can be discovered in AIH. This study highlighted three existing drugs, anifrolumab, sirolimus, and G-CSF, as potentially promising therapies for AIH. Future studies using single cell RNA-Seq analysis of whole blood from AIH patients as well as studies of liver biospecimens may shed more light on these results. By applying current genomic technology to biospecimens from AIH patients, we hope to provide answers to the many remaining questions in this challenging disease.

## Supporting information

S1 Fig Flow chart depicting algorithm for determining fibrosis status of AIH patient. (TIF)

S2 Fig PCA plot of variance stabilizing transformed gene counts colored by A. age bracket of patient, B. race/ethnicity of patient, and C. library preparation batch for patient sample.
(TIF)

S3 Fig Top ten most significant canonical pathways related to differential gene expression of treatment naïve AIH patients compared to healthy controls. (TIF)

S1 Table Most differentially expressed genes in AIH patients compared to healthy volunteers. (XLSX)

S2 Table List of all upstream regulators identified by pathway analysis for treatment-naïve patients compared to healthy controls. Republished from Ingenuity Pathway Analysis under a CC BY license, with permission from Qiagen, original copyright 2019.
(XLS)

S3 Table Top 12 hub genes correlated with AIH cirrhosis. (XLSX)

## Abbreviations

AIH: autoimmune hepatitis
RNA-seq: RNA sequencing
GRACE: Genetic Repository of Autoimmune Liver Diseases and Contributing Exposures
rRNA: ribosomal RNA
