# Supplementary figures and images for: Transcriptomic profiling of blood from autoimmune hepatitis patients reveals potential mechanisms with implications for management
Source: PLoS One. 2022 Mar 21;17(3):e0264307. doi: 10.1371/journal.pone.0264307 (PMC8936448; doi:10.1371/journal.pone.0264307)

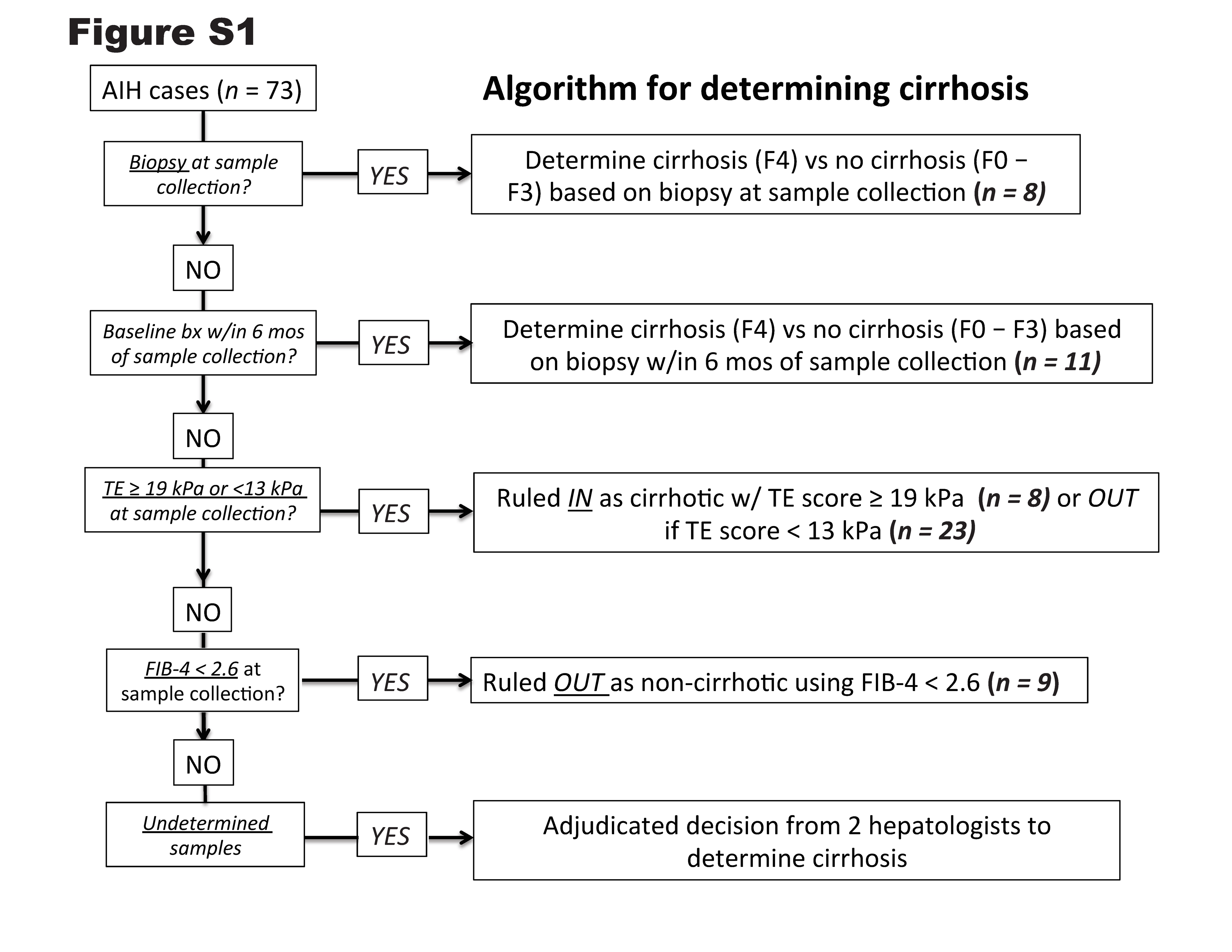

Supplement: S1 Fig — (TIF) [file pone.0264307.s001.tif]

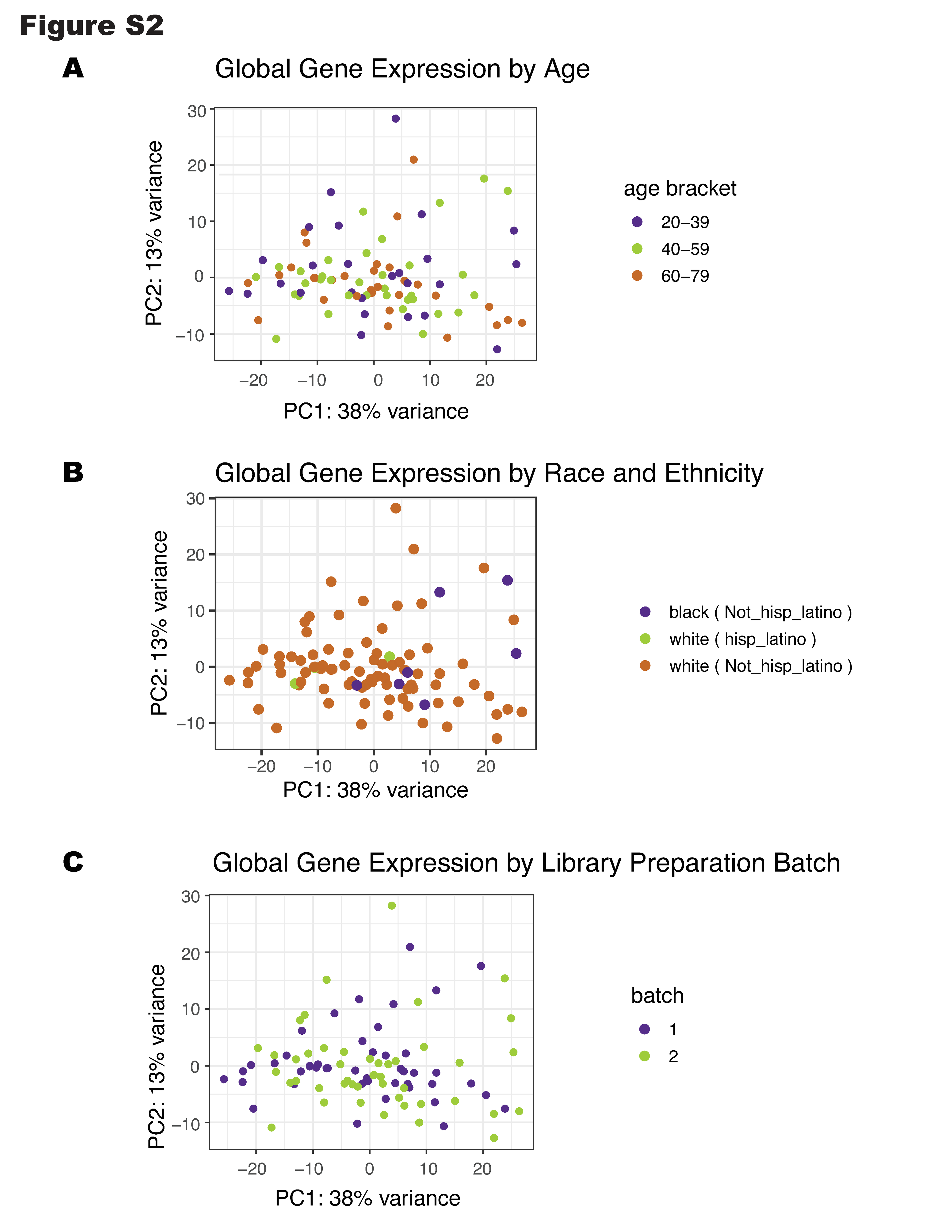

Supplement: S2 Fig — PCA plot of variance stabilizing transformed gene counts colored by A. age bracket of patient, B. race/ethnicity of patient, and C. library preparation batch for patient sample. (TIF) [file pone.0264307.s002.tif]

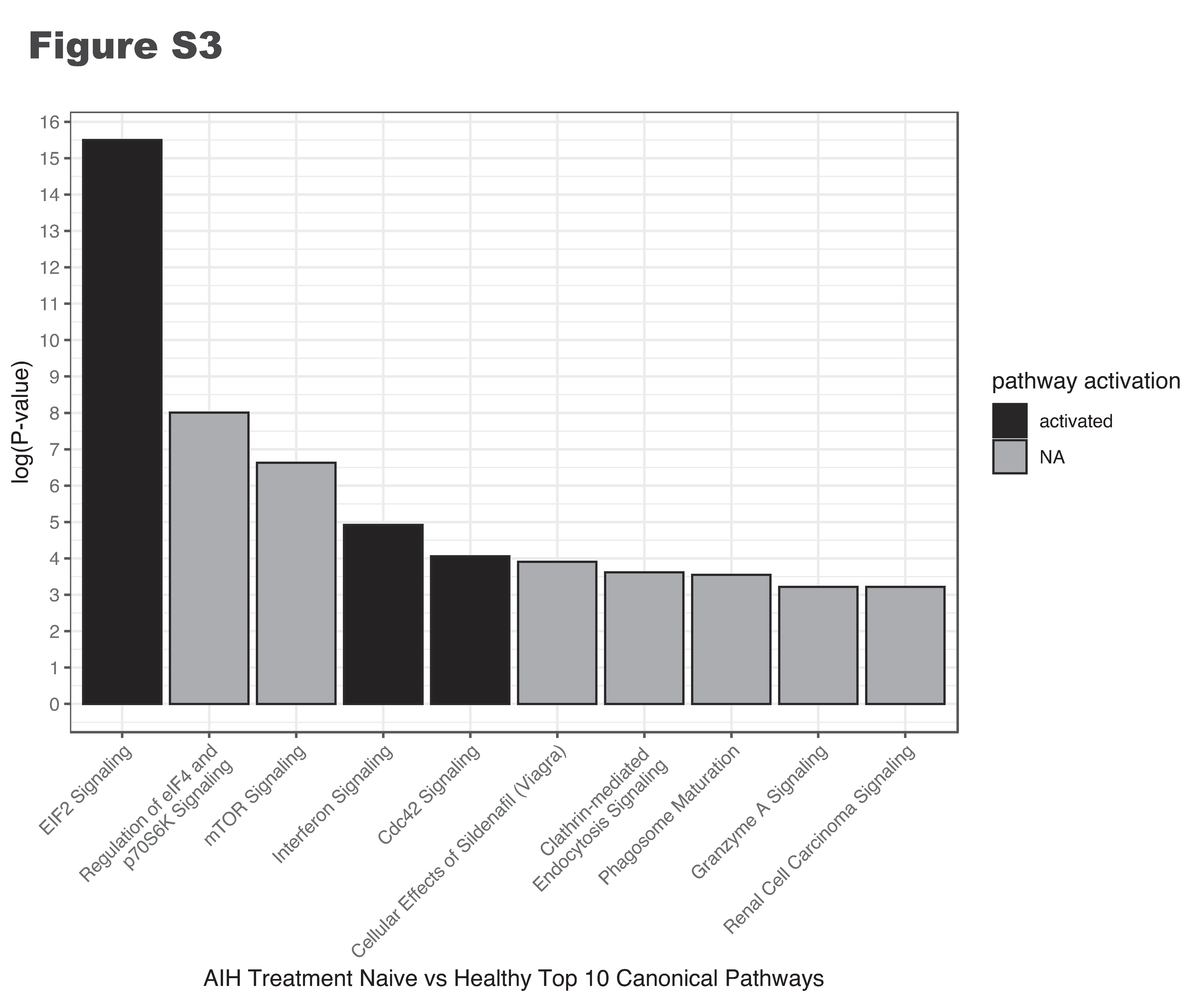

Supplement: S3 Fig — (TIF) [file pone.0264307.s003.tif]
