# Supplementary material for: Transcriptomic profiling of blood from autoimmune hepatitis patients reveals potential mechanisms with implications for management
Source: PLoS One. 2022 Mar 21;17(3):e0264307. doi: 10.1371/journal.pone.0264307 (PMC8936448; doi:10.1371/journal.pone.0264307)
Supplement: Attachment — Submitted filename: Response to Reviewers 12-20-20.docx [file pone.0264307.s007.docx]

Dear PLOS ONE Editors:

Thank you for sharing the reviewers’ comments to our previous submission PONE-D-20-22091. Here are our point by point responses:

Reviewer #1’s Comments

1) Results. Figure 1 and Table 2. The authors indicate that 9 or fewer genes could differentiate between AIH and controls. This is very exciting. The authors should perform PCA on covariance of the genes to determine if there are indeed clusters that vary together. This is often performed to justify that a handful of genes can serve as biomarkers for the whole.

*We performed PCA on covariance of the genes and didn't find obvious clusters when looking at cases vs. controls. However, we did see hierarchical clustering of genes when we focused on the 1000 most highly expressed genes in the dataset (see Figure 1B). We modified the Results to clarify this point.*

2) The discussion of the potential that pegivirus is a little too premature given the paucity of representation in this small cohort. The subtitle is a little misleading. 5 of 75 patients is not “several”.

*We modified the subtitle to “handful” and reworded our discussion to more conservatively describe the possible role of pegivirus.*

3) Page 15, lines 328-329. The analysis of these results (i.e., movement of T cells) is speculative and should be reserved for the Discussion.

*We moved this sentence to the Discussion.*

4) As mentioned above, while the authors find some interesting results, they need to be validated. It is understood that a validation cohort is beyond the scope of the current work, however, the authors could determine a preliminary AUROC of training on part of the cohort, and validation on the second part.

*We clarified our discussion to address the need for validation in a separate cohort, which is being developed.*

Reviewer #2’s Comments

1) “Controls were healthy volunteers” – Please clarify if these were randomly recruited patients from in/outpatient settings or if they were age/sex-matched or matched on other criteria to the serially recruited AIH cases. It appears from the results that these were unmatched. How were the controls chosen?

*We clarified this section to say, “The controls were randomly recruited from the community at Autoimmune Hepatitis Association conferences and other events. They were screened for liver disease through self-reported history and through blood tests of liver enzymes.*

2) The sentence “In addition, low count gene filter was applied to remove genes which had fewer than 10 reads in less than 20% of the samples”. This is a little confusing. Iʼm assuming you removed genes that had fewer than 10 reads in 20% or more of the samples?

*We clarified this sentence to read, “In addition, a filter for genes with low counts was applied to remove genes that had fewer than 10 reads in 20% or more of the samples.*

3) The Differential Gene Expression section appears to be generic and nonspecific to this analysis. Please clearly define your variable of interest (Iʼm assuming AIH vs. controls). Please also include any data cleaning/pre-processing steps (e.g., norm factors between samples)

*We rewrote this section to include pre-processing steps and define variables of interest.*

4) For the pathway enrichment analysis, was an adjusted p-value threshold not used? Using log-fold change alone could include genes with extremely high variance and unreliable results

*We clarified the Methods section on Pathway Enrichment Analysis to state that we did not use a p-value cutoff. For the analysis of AIH cases vs. healthy controls, the pathway results were similar with or without a p-value cutoff. For the analysis of treatment-naïve AIH vs. healthy controls, use of a p-value cutoff made pathway analysis impossible.*

5) Though the first two principal components donʼt show a strong gradient by sex, there is still concern that sex could be confounding these results. Since only 51% of the variance is explained by PC1 and PC2, it may be prudent to include a few more principal component scatterplots until a higher percent of variance (~ 80%?) is explained. If you find that sex is clearly delineated in a top PC, then it may be good to adjust for sex in many of the other analyses; otherwise, note this as a limitation in the discussion.

*We added a sentence to the discussion: “While sex is not clearly delineated in PC1 or PC2, this does not rule out the possibility that sex could be a minor confounder.”*

6) Many of the genes in the random forest (using standard deviation multiplicative factor of 1) are very unstable (appear in < 40% of seeds), it might be reasonable to exclude these. Or to perform a clustering prior to using the random forest approach to increase stability. I worry that with increasing sample size many of these effects would be spurious.

*We added a sentence to the Discussion, “However, we recognize that many of these genes appear in less than half of seeds and these may not be stable predictors when applied to validation datasets. We acknowledge that our predictive power is greatly limited by the small size of this pilot cohort and emphasize that this algorithm does not have clinical significance without further testing and validation.”*

7) “We used an unbiased approach to perform pathway analysis” – Itʼs unclear what “unbiased”means in this situation. This should be a biased approach in that you are using the label of AIH vs control to determine active pathways.

*We removed the word “unbiased from this sentence.*

8) Lowest p-value does not correspond to “most differentially regulated”. A better way of saying this is “most likely to be differentially regulated (by p-value)”.

*We edited the sentence as Reviewer #2 suggested.*

9) The heading “Gene expression correlates with poor outcomes in AIH” however a majority of these phenotypes are not future outcomes but present disease. This title could better reflect this.

*We changed the heading to “Gene expression correlates with fibrosis stage in AIH”*

10) Is there an explanation why Differences in absolute CD8 signal should be present in the healthy vs partial response contrast but not the healthy vs complete response contrast? As this is an exploratory study, it might be nice to include the actual p-value. Second, this sentence is unclear,

“AIH subjects with partial response to therapy (n =18) had a lower CD8 T cell signature in the periphery (Figure 4A), which was independent of steroid therapy (Figure 4B).” AIH subjects with partial response to therapy are being compared to which group in this sentence? Please clarify. Figure 4B appears to be Partial vs complete response in those that did not have steroids. In addition, you should indicate the p-value for the comparison between complete and partial responses in the full cohort. Is this not significant when you donʼt remove the patients on steroids, because then “independent of steroids” is misleading.

*We included the exact p-values in the Results text. We clarified the sentence to read, “AIH subjects with partial response to therapy (n=18) had a lower CD8 T cell signature in the periphery compared to healthy controls (p=0.05) and compared to complete responders (p=0.04) (Figure 4A). After excluding patients on corticosteroids from the analysis, partial responders still had a lower CD8 T cell signature than complete responders (p=0.05, Figure 4B).”*

*We modified the Discussion to include a possible explanation of our findings, “While complete responders may have a CD8 response that is “restored” or closer in character to healthy controls given their treatment response, partial responders may still have active AIH disease activity, i.e. a CD8 signature more similar to active AIH.”*

11) In the discussion, it needs to be clearly stated as a limitation, that the genes selected by the random forest method have not been validated for clinical diagnosis. For this, you would need to validate the model in an independent validation cohort or at least perform a cross-validation to get some sense of generalizability of the selected genes. I would also remove that there is a “12-gene signature” from the abstract as this implies a predictive signature, which was not validated in this study.

*We modified the discussion to acknowledge that the sample size for this pilot was relatively small and therefore the gene set identified by the random forest algorithm cannot be used clinically without further testing and validation. We removed “12-gene signature” from the abstract. We replaced it with, “The gene expression data and clinical metadata were used to determine 12 genes that are significantly associated with advanced fibrosis in AIH.”*

12) The authors intend to make their data publicly available; however, I did not see a GitHub link nor an SRA number yet. Please add this to the manuscript.

*We added to Results, “Full data set available at github link* [*https://github.com/czbiohub/AIH-Project*](https://github.com/czbiohub/AIH-Project)*”*

13) Grammar/Typos: There are a few instances with missing references “(reference here)” or “(ref here)” – please go through and correct these “This analysis was performed using the Weighted Gene Correlation Network Analysis algorithm in the R…” -> This analysis was performed using the Weighted Gene Correlation Network Analysis algorithm in R. “Of subjects on AIH therapy, 42% were on a regimen that included corticosteroids.” “Relevant metadata classifying samples based on patient status” -> “Relevant metadata included patient status, steroid exposure, and fibrosis groups to classify samples” “The strongest signal of genes differentially expressed in AIH compared to healthy ¬controls was that of interferon activation.”

*In Methods section on DASH, we included the reference PMID: 269447.*

*Under Methods, Clinical Cohort, we added the reference PMID: 30617453 for the GRACE Cohort.*

*We removed “ref here” from the sentence about PRICESeq filter.*

*We deleted “the” so the sentence on WGCNA reads, “This analysis was performed using the Weighted Gene Correlation Network Analysis algorithm in R.”*

*We changed “was” to “were” in the sentence, “Of subjects on AIH therapy, 42% were on a regimen that included corticosteroids.”*

*We changed the caption for Figure 1B to read, “Relevant metadata included patient status, steroid exposure, and fibrosis groups to classify samples.”*

*We added the word “controls” so that the sentence in the Discussion now reads, “The strongest signal of genes differentially expressed in AIH compared to healthy controls was that of interferon activation.”*

Sincerely,

The AIH Transcriptomics Pilot Research Team
