# Supplementary material for: Transcriptomic profiling of blood from autoimmune hepatitis patients reveals potential mechanisms with implications for management
Source: PLoS One. 2022 Mar 21;17(3):e0264307. doi: 10.1371/journal.pone.0264307 (PMC8936448; doi:10.1371/journal.pone.0264307)
Supplement: Peer review history

# Author comment: Author response to previous submission

**DOI:** 10.1371/journal.pone.0264307.r001

22 Dec 2020

Attachment Submitted filename: Response to Reviewers 12-20-20.docx Click here for additional data file.

# Peer review: Decision Letter 0

**DOI:** 10.1371/journal.pone.0264307.r002

**Authors:** Frederic Rieux-Laucat

10 Jun 2021

PONE-D-20-39992

Transcriptomic Profiling of Blood from Autoimmune Hepatitis Patients Reveals Potential Mechanisms and Management Options

PLOS ONE

Dear Dr. Tana,

Thank you for submitting your manuscript to PLOS ONE. After careful consideration, we feel that it has merit but does not fully meet PLOS ONE’s publication criteria as it currently stands. Therefore, we invite you to submit a revised version of the manuscript that addresses the points raised during the review process.

See reviewers comments and concerns below.

Please submit your revised manuscript by Jul 25 2021 11:59PM. If you will need more time than this to complete your revisions, please reply to this message or contact the journal office at plosone@plos.org. When you're ready to submit your revision, log on to https://www.editorialmanager.com/pone/ and select the 'Submissions Needing Revision' folder to locate your manuscript file.

Please include the following items when submitting your revised manuscript:

- A rebuttal letter that responds to each point raised by the academic editor and reviewer(s). You should upload this letter as a separate file labeled 'Response to Reviewers'.
- A marked-up copy of your manuscript that highlights changes made to the original version. You should upload this as a separate file labeled 'Revised Manuscript with Track Changes'.
- An unmarked version of your revised paper without tracked changes. You should upload this as a separate file labeled 'Manuscript'.

If you would like to make changes to your financial disclosure, please include your updated statement in your cover letter. Guidelines for resubmitting your figure files are available below the reviewer comments at the end of this letter.

If applicable, we recommend that you deposit your laboratory protocols in protocols.io to enhance the reproducibility of your results. Protocols.io assigns your protocol its own identifier (DOI) so that it can be cited independently in the future. For instructions see: http://journals.plos.org/plosone/s/submission-guidelines#loc-laboratory-protocols. Additionally, PLOS ONE offers an option for publishing peer-reviewed Lab Protocol articles, which describe protocols hosted on protocols.io. Read more information on sharing protocols at https://plos.org/protocols?utm_medium=editorial-email&utm_source=authorletters&utm_campaign=protocols.

We look forward to receiving your revised manuscript.

Kind regards,

Frederic Rieux-Laucat, PhD

Academic Editor

PLOS ONE

Journal Requirements:

When submitting your revision, we need you to address these additional requirements.

1. Please ensure that your manuscript meets PLOS ONE's style requirements, including those for file naming. The PLOS ONE style templates can be found at

https://journals.plos.org/plosone/s/file?id=wjVg/PLOSOne_formatting_sample_main_body.pdf and

https://journals.plos.org/plosone/s/file?id=ba62/PLOSOne_formatting_sample_title_authors_affiliations.pdf

2. We note that you have stated that you will provide repository information for your data at acceptance. Should your manuscript be accepted for publication, we will hold it until you provide the relevant accession numbers or DOIs necessary to access your data. If you wish to make changes to your Data Availability statement, please describe these changes in your cover letter and we will update your Data Availability statement to reflect the information you provide.

3. Please upload a copy of Supporting Information Tables 1-3 which you refer to in your text on pages 26 and 27.

[Note: HTML markup is below. Please do not edit.]

Reviewers' comments:

Reviewer's Responses to Questions

**Comments to the Author**

1. Is the manuscript technically sound, and do the data support the conclusions?

The manuscript must describe a technically sound piece of scientific research with data that supports the conclusions. Experiments must have been conducted rigorously, with appropriate controls, replication, and sample sizes. The conclusions must be drawn appropriately based on the data presented. 

Reviewer #1: Yes

Reviewer #2: No

Reviewer #3: Yes

**********

2. Has the statistical analysis been performed appropriately and rigorously? 

Reviewer #1: Yes

Reviewer #2: No

Reviewer #3: Yes

**********

3. Have the authors made all data underlying the findings in their manuscript fully available?

The PLOS Data policy requires authors to make all data underlying the findings described in their manuscript fully available without restriction, with rare exception (please refer to the Data Availability Statement in the manuscript PDF file). The data should be provided as part of the manuscript or its supporting information, or deposited to a public repository. For example, in addition to summary statistics, the data points behind means, medians and variance measures should be available. If there are restrictions on publicly sharing data—e.g. participant privacy or use of data from a third party—those must be specified.

Reviewer #1: Yes

Reviewer #2: Yes

Reviewer #3: Yes

**********

4. Is the manuscript presented in an intelligible fashion and written in standard English?

PLOS ONE does not copyedit accepted manuscripts, so the language in submitted articles must be clear, correct, and unambiguous. Any typographical or grammatical errors should be corrected at revision, so please note any specific errors here.

Reviewer #1: Yes

Reviewer #2: Yes

Reviewer #3: Yes

**********

5. Review Comments to the Author

Please use the space provided to explain your answers to the questions above. You may also include additional comments for the author, including concerns about dual publication, research ethics, or publication ethics. (Please upload your review as an attachment if it exceeds 20,000 characters)

Reviewer #1: Authors Michele May-Sien Tana et al performed bulk RNA sequencing on whole blood in 75 AIH patients and 25 healthy volunteers.

They identified a set of genes which are differentially expressed in AIH patients as compared to healthy controls, including the interferon signaling pathway.

They also compared gene expression modulation in patients treatment-naive patients as compared to healthy controls in order to identify potential target of well-known drugs. The data suggested that Sirolimus or GM-CSF could be a potential treatment in AIH patient. This study need to be expand and validated in a larger cohort.

Major comments:

1. Concerning the healthy controls, you need to precise that they signed an informed consent prior to be enrolled in the study.

2. The supplemental Tables are missing.

3. For the pathway enrichment analysis: why did the authors choose not to use any p-value cut-off (line183)?

Minor comments:

1. The use of Sirolimus as a potential drug can be discussed in relation to the modulation of the mTOR pathway depicted in figure S3

2. Some typos remain in the manuscript. References should be placed before the full stop at the end of each sentence.

3. These analyses could benefit from comparison of the same explorations on liver biopsies in parallel.

In total, this work paved the way of a better understanding of the physiopathology of AIH, and need to be extend and validated in a larger cohort.

Reviewer #2: As stated by the authors, there is a need in AIH for easily testable biomarkers to help diagnosis and prognosis in AIH and transcriptomic profiling seems indeed a good approach to answer this need. As it stands, the presence of an IFN response and the identification of some genes as potential biomarkers could represent some interesting findings but they are weakened by the lack of validation. The top 12 genes described could be easily tested on other patients and/or on the same patient by quantitative PCR and/or microarrays to validate their significance. IFN signatures could also be investigated using other methods and finally an immune phenotyping of the whole blood seems to be required to validate their in-silico prediction about CD8 T cells.

- Line 2: Title : “management Options” makes the title quite difficult to understand out of context

- Line 99: as not every patient received a liver biopsy to estimate fibrosis stage are there any bias in gene expression profiles based on how cirrhosis was determined ( grops can be established based on the different options listed in fig S1

- Line 182: as already stated by another reviewer, the absence of filtering based on adjusted p-value cutoff seems rather hazardous in particular when it comes to identify genes and pathways potentially used for diagnosis purpose. Answer already provided to reviewer#2 is not clear and the fact that IPA did not return any pathways when using an adjusted p-value regarding treatment-naïve AIH vs healthy is rather concerning. Could authors elaborate more their choice to continue analyzing data without adjusted p-value cutoff?

- Line 223: Type 1, Type 2 and other types of AIH are not clearly defined nor in Introduction, material and methods and those classifications do not appear in Table 1

- Line 238: There is on conclusion drawn from figure 1B which is rather surprising? Does the clustering make sense for the different groups in Y axis for example? Commenting such clustering is rather important as a quality control to ensure that everything is correct and that the analyses can be pushed further.

- Same comment for figure 1C: if there is nothing to be said about fig 1C, then it should be moved to sup fig.

- Line 244: 249 DEG only when comparing cases to controls seem a rather small number based on the fact that the analysis was done on whole blood. It could reflect a quite heterogeneous representation among “cases” or the need to do a more refine analysis in cell subtypes. Could the author comments on this small number of DEG in cases vs controls?

- Line 245: Could the authors explain how parameters were chosen and incremented regarding the random forest approach?

- Line 256: pathway analysis was performed on what? I guess it is on the 249 DEG but not really clear ? the 9 genes found by random forest modeling?

- Line 265: How many genes differentially expressed between treatment-naïve and healthy controls? Why does the authors did not comment on the appearance of other pathways when comparing treatment naïve to healthy subject? EIF2, EIF4 signaling, mTOR? They could all be potentially interesting.

- Line 270: upstream regulator analysis was performed on the 249 DEG? The number of significant pathways seems this time really high for such a low number of genes.

- Line 290: conclusion of this paragraph should rather be that using the pathogen detection platform, no evidence of viral presence was detected in the majority of samples and could not therefore explain the increase of IFN signaling pathways.

- Line 306: Can inverse correlation be interesting in those type of analyses? Clusters 2,3,4 and 5 have quite a strong inverse correlation. Could authors comments on those?

- Line 319: Could the authors define gene significance?

- Line 324: As stated by the authors the segregation of individual with AIH based on cirrhosis status is only partial. If a signature score combining the expression of those top genes is used, could the authors obtained a better segregation?

- Line 337: Using a computational method alone to assess contribution of individual immune population from whole blood bulk RNA-seq data, seems a rather risky approach. Authors should confirm their predictions by looking at immune cells distribution in the blood through classical immunophenotyping approach (flow cytometry, CyTOF for example).

- What are the results obtained with CIBERSORT regarding the other cell types? This would be particularly useful to understand how reliable the results of the deconvolution are?

- Line 402: Sirolimus is a treatment, so author should be careful of the words used to explain Sirolimus as an inhibited upstream regulator. All regulators not linked with transcription factors should have been excluded from their upstream analyses.

- Line 405: conclusions made by the authors would need further supports from their data. RICTOR changes of expressions in all their samples? Predicted target changes of expression?

- Line 476 and 477: those lines should be removed from the discussion as theire is no correlation between pegivirus presence and AIH.

Reviewer #3: - The authors performed RNAseq in the peripheral blood of patients with auto immune hepatitis (AIH) to gain insight into the pathogenesis of this disease.

- This study is very exciting and in my opinion, figure 2A is on of the most interesting finding of the study. It compares the top 10 canonial pathways in patients with AIH vs healthy subjects. One limitation is that all AIH patients are included regardless of the treatment they received, and this can of course interfere with the pathways and be a confounding factor. When the authors perform this analysis only on patients naive of treatment (n=5), they can only draw a conclusion on interferon signaling and not on the other caonical pathways due to lack of power.

- Table 1: Treatment type. I would like the authors to give more detail (in the table or in the text) about treatment type. Treatment received is a main confounding factor in this study, therefore, it is important to have maximum information about treatment received.

-> "Other treatment": I would like to know the name of these treatments

-> "Off treatment": cause ? (remission? Non observance ? )

-> " Steroids". Usually, AIH treatment consists of the association of steroids AND another drug (usually azathioprine). Patients are not often treated with steroids only. I would like more information about the treatment associated with steroids (if any) for each of the 28 patients on steroids.

- Figure 4: Treatment received can explain a lower CD8 count. Excluding patients on steroids (and not on other treatments that may have an impact of CD8 count such as I think azathioprine) is not enough (Figure 4 B).I think this should be mentionned in the discussion. If you want to have results more interpretable, you should repeat this analysis but for a given treatment (ie: compare count of CD8+ of patients on steroids only (or another drug but the same one), to show if there is a difference between complete and partial response).

- 384 "and that pegivirus is more prevalent in patients with liver transplant that hepatectomy patients" -> I do not understand what the authors means by "hepatectomy patients" (hepatectomy means the removal of one's liver).

- 432 "in many patients with early cirrhosis fibrosis stage in only apparent ...". I am not sure to understand what the authors mean by "early cirrhosis".

- 433: I would like to point out that fibroScan and shear wave devices are recognised and validated non invasive methods for the diagnosis of cirrhosis.

- 435 "and advanced disease". I am not sure to understand what the authors means by "advanced disease".

- Table 2 and 3A are blurry

**********

6. PLOS authors have the option to publish the peer review history of their article (what does this mean?). If published, this will include your full peer review and any attached files.

If you choose “no”, your identity will remain anonymous but your review may still be made public.

**Do you want your identity to be public for this peer review?** For information about this choice, including consent withdrawal, please see our Privacy Policy.

Reviewer #1: No

Reviewer #2: No

Reviewer #3: **Yes: **Claire MAYER

[NOTE: If reviewer comments were submitted as an attachment file, they will be attached to this email and accessible via the submission site. Please log into your account, locate the manuscript record, and check for the action link "View Attachments". If this link does not appear, there are no attachment files.]

While revising your submission, please upload your figure files to the Preflight Analysis and Conversion Engine (PACE) digital diagnostic tool, https://pacev2.apexcovantage.com/. PACE helps ensure that figures meet PLOS requirements. To use PACE, you must first register as a user. Registration is free. Then, login and navigate to the UPLOAD tab, where you will find detailed instructions on how to use the tool. If you encounter any issues or have any questions when using PACE, please email PLOS at figures@plos.org. Please note that Supporting Information files do not need this step.

# Author comment: Author response to Decision Letter 0

**DOI:** 10.1371/journal.pone.0264307.r003

28 Jul 2021

Reviewer #1: Authors Michele May-Sien Tana et al performed bulk RNA sequencing on whole blood in 75 AIH patients and 25 healthy volunteers.

They identified a set of genes which are differentially expressed in AIH patients as compared to healthy controls, including the interferon signaling pathway.

They also compared gene expression modulation in patients treatment-naive patients as compared to healthy controls in order to identify potential target of well-known drugs. The data suggested that Sirolimus or GM-CSF could be a potential treatment in AIH patient. This study need to be expand and validated in a larger cohort.

Major comments:

1. Concerning the healthy controls, you need to precise that they signed an informed consent prior to be enrolled in the study.

We specified that the healthy controls and AIH cases had also provided informed consent. 

2. The supplemental Tables are missing.

We uploaded the supplemental tables.

3. For the pathway enrichment analysis: why did the authors choose not to use any p-value cut-off (line183)?

We have clarified the Methods and modified our Discussion to explain this choice.

“Differential gene expression results were imported into Ingenuity Pathway Analysis software (Qiagen, Redwood City, CA, USA). Genes were considered for entry into the analysis if they had an absolute log fold change greater than 0.6, but no adjusted p-value cutoff was required.”

“With the small number of treatment-naive patients, our analysis of pathways activated or inhibited in this group could not use a p-value cutoff for genes considered in the analysis and was thus exploratory only. For the analysis of AIH cases vs. healthy controls, the pathway results were similar with or without a p-value cutoff for considered genes.”

Minor comments:

1. The use of Sirolimus as a potential drug can be discussed in relation to the modulation of the mTOR pathway depicted in figure S3

We referenced Figure S3 and the mTOR pathway in the part of the Discussion about sirolimus.

“Sirolimus is a macrolide drug that inhibits mTOR, a protein that controls the proliferation and survival of activated lymphocytes. Indeed, mTOR signaling was one of the most significant pathways in our focused analysis of treatment-naïve patients vs. healthy controls (Figure S3).”

2. Some typos remain in the manuscript. References should be placed before the full stop at the end of each sentence.

We fixed the typos and placed superscripts before the full stop of each sentence.

3. These analyses could benefit from comparison of the same explorations on liver biopsies in parallel.

We added liver biopsies as a future direction in the Discussion:

“further efforts to expand longitudinal cohorts that include AIH patients, clinical data, and blood and liver biospecimens are currently underway”

In total, this work paved the way of a better understanding of the physiopathology of AIH, and need to be extend and validated in a larger cohort.

Reviewer #2: As stated by the authors, there is a need in AIH for easily testable biomarkers to help diagnosis and prognosis in AIH and transcriptomic profiling seems indeed a good approach to answer this need. As it stands, the presence of an IFN response and the identification of some genes as potential biomarkers could represent some interesting findings but they are weakened by the lack of validation. The top 12 genes described could be easily tested on other patients and/or on the same patient by quantitative PCR and/or microarrays to validate their significance. IFN signatures could also be investigated using other methods and finally an immune phenotyping of the whole blood seems to be required to validate their in-silico prediction about CD8 T cells.

- Line 2: Title : “management Options” makes the title quite difficult to understand out of context

We changed the title to “Transcriptomic Profiling of Blood from Autoimmune Hepatitis Patients Reveals Potential Mechanisms with Implications for Management.”

- Line 99: as not every patient received a liver biopsy to estimate fibrosis stage are there any bias in gene expression profiles based on how cirrhosis was determined ( grops can be established based on the different options listed in fig S1

We acknowledged this potential for bias in the Discussion:

“We did not have liver biopsy data on all patients but developed an algorithm for determining if patients had cirrhosis, which raises the possibility of bias in our analysis.”

- Line 182: as already stated by another reviewer, the absence of filtering based on adjusted p-value cutoff seems rather hazardous in particular when it comes to identify genes and pathways potentially used for diagnosis purpose. Answer already provided to reviewer#2 is not clear and the fact that IPA did not return any pathways when using an adjusted p-value regarding treatment-naïve AIH vs healthy is rather concerning. Could authors elaborate more their choice to continue analyzing data without adjusted p-value cutoff?

We did not use a p-value threshold for genes to include in the pathway analysis, but the software did assign p-values to individual pathways that it identified. The number of treatment-naïve AIH patients in the cohort was small (n=5) because once a diagnosis is made, physicians start therapy rapidly. Hence, most patients donated blood after treatment had been initiated. Therefore, we did not have the statistical power to generate low p-values for many genes. However, we wanted to include differentially expressed genes (log-fold 0.6 or greater) in the pathway analysis. We acknowledge these limitations in the Discussion, and state that this was an exploratory pilot study.

Please see our response to Reviewer #1’s major comment #3 about line 183.

“Differential gene expression results were imported into Ingenuity Pathway Analysis software (Qiagen, Redwood City, CA, USA). Genes were considered for entry into the analysis if they had an absolute log fold change greater than 0.6, but no adjusted p-value cutoff was required.”

“With the small number of treatment-naive patients, our analysis of pathways activated or inhibited in this group could not use a p-value cutoff for genes considered in the analysis and was thus exploratory only. For the analysis of AIH cases vs. healthy controls, the pathway results were similar with or without a p-value cutoff for included genes.”

- Line 223: Type 1, Type 2 and other types of AIH are not clearly defined nor in Introduction, material and methods and those classifications do not appear in Table 1

We added definitions for Type 1 and Type 2 AIH in the Methods:

“Autoimmune hepatitis type was determined through chart review. Type 1 was defined by positivity of antinuclear, anti-smooth muscle, or anti-actin antibodies. Type 2 was defined by antibodies to LKM-1 or LC-1. Cases following exposure to an offending drug with the ability to wean immunosuppression was classified as drug-induced AIH.”

- Line 238: There is on conclusion drawn from figure 1B which is rather surprising? Does the clustering make sense for the different groups in Y axis for example? Commenting such clustering is rather important as a quality control to ensure that everything is correct and that the analyses can be pushed further.

We enhanced the Results to comment on the clustering in Figure 1B:

“Unsupervised clustering of the 1000 most variably expressed genes in the dataset did not entirely separate samples based on diagnosis, treatment, or fibrosis stage.”

- Same comment for figure 1C: if there is nothing to be said about fig 1C, then it should be moved to sup fig.

We enhanced the Results to comment on the clustering in Figure 1C:

“Unsupervised clustering analysis on all gene counts did not reveal grouping of samples by demographic factors such as age, sex, and race, nor by RNA preparation batch (Figure 1C, Figure S2).”

- Line 244: 249 DEG only when comparing cases to controls seem a rather small number based on the fact that the analysis was done on whole blood. It could reflect a quite heterogeneous representation among “cases” or the need to do a more refine analysis in cell subtypes. Could the author comments on this small number of DEG in cases vs controls?

We added comments on the number of differentially expressed genes to the Discussion:

“We found 249 genes that were significantly differentially expressed in the whole blood of AIH patients compared to healthy controls. This relatively small number of differentially expressed genes could be related to the fact that most blood samples were drawn from AIH patients after the initiation of therapy, heterogeneity among cases and/or, or perhaps the need to focus on certain cell types within whole blood.”

- Line 245: Could the authors explain how parameters were chosen and incremented regarding the random forest approach?

We used varSelRF with a large number of trees as that has been shown to be optimal for the random forest algorithm [Breiman, Machine Learning, 2001, https://link.springer.com/article/10.1023/A:1010933404324]. In brief, we used a large enough forest to increase the consistency of the results. The OOB error is the parameter to which the RF is more sensitive to and that is what is actually minimized during the process of variable elimination. The reason for 2000 iterations is to have a robust estimate of the variable importance. Random forests do not overfit in general and there is no need to perform cross-validation because that is built in the algorithm. For a more complete explanation please refer to the notes of the original implementation of the algorithm (https://www.stat.berkeley.edu/~breiman/RandomForests/cc_home.htm). We have added the Breiman, Machine Learning 2001 paper as a reference in the manuscript.

- Line 256: pathway analysis was performed on what? I guess it is on the 249 DEG but not really clear ? the 9 genes found by random forest modeling?

Pathway analysis was performed on all protein-coding genes with an absolute log fold change (between the groups being compared) greater than 0.6. We clarified this in the Methods.

“Genes were considered for entry into the analysis if they had an absolute log fold change greater than 0.6 between the two groups of interest (AIH cases vs. healthy controls, or treatment-naïve AIH vs. healthy controls)”

- Line 265: How many genes differentially expressed between treatment-naïve and healthy controls? Why does the authors did not comment on the appearance of other pathways when comparing treatment naïve to healthy subject? EIF2, EIF4 signaling, mTOR? They could all be potentially interesting.

We added more commentary on activated pathways to the Results section.

“Analysis further identified several additional pathways of interest, for example, myeloid signaling, as indicated by activation of triggering receptor expressed on myeloid cells 1 (TREM1) and inflammasome pathways, but inhibition of liver X receptor/retinoid X receptor (LXR/RXR) activation (Fig. 2A)”

- Line 270: upstream regulator analysis was performed on the 249 DEG? The number of significant pathways seems this time really high for such a low number of genes.

As above, we clarified in the Methods that IPA (Ingenuity Pathway Analysis) used all protein-coding genes with a log-fold change of .6 or greater between AIH cases and healthy controls or tx-naïve AIH and healthy.

- Line 290: conclusion of this paragraph should rather be that using the pathogen detection platform, no evidence of viral presence was detected in the majority of samples and could not therefore explain the increase of IFN signaling pathways.

We modified the paragraph heading per the reviewer’s suggestion:

“Pathogen detection platform reveals infection with pegivirus in a handful of samples, but cannot fully explain the activation of interferon signaling in AIH compared to healthy controls”

- Line 306: Can inverse correlation be interesting in those type of analyses? Clusters 2,3,4 and 5 have quite a strong inverse correlation. Could authors comments on those?

Yes, inverse correlation can also be significant in WGCNA. We commented on gene module 5 in the Results.

“Conversely, module 5 was significantly inversely correlated with clinical variables related to advanced fibrosis (fibroscan score, FIB-4 score, cirrhosis, decompensation, and need for transplant).”

- Line 319: Could the authors define gene significance?

Yes, the Methods section was updated to clarify what the p-value represents as below:

“To identify hub genes, we used a gene significance cutoff of 0.5 and a measure of centrality within the module, module membership, at a cutoff of 0.7. To assess the strength of the correlation between genes of interest and individual modules, gene significance for the genes in the module were compared to module membership using univariate regression, and correlation p-values were generated.”

- Line 324: As stated by the authors the segregation of individual with AIH based on cirrhosis status is only partial. If a signature score combining the expression of those top genes is used, could the authors obtained a better segregation?

Yes, a goal for future directions is to obtain additional patient samples to create a validation cohort where we can test identified signatures, on a more selected set of top genes in order to improve segregation of phenotypes and assess whether these genes may have predictive value. Unfortunately, we did not have the sample size for this in our current study. 

- Line 337: Using a computational method alone to assess contribution of individual immune population from whole blood bulk RNA-seq data, seems a rather risky approach. Authors should confirm their predictions by looking at immune cells distribution in the blood through classical immunophenotyping approach (flow cytometry, CyTOF for example).

Yes, this would be ideal. Unfortunately, the whole blood specimens used in this study were collected and frozen in Paxgene tubes in order to preserve intact RNA extraction, which does not allow for analysis of individual cells / cell surface proteins by flow cytometry/CyTOF. A goal for future study would be to perform cell-type specific analysis (eg flow cytometry, scRNA Seq, etc). We added to the discussion that classical immunophenotyping will be a future step.

“However, further efforts to expand longitudinal cohorts that include AIH patients, clinical data, and blood and liver biospecimens are currently underway, and classical immunophenotyping assays are planned.”

- What are the results obtained with CIBERSORT regarding the other cell types? This would be particularly useful to understand how reliable the results of the deconvolution are?

Below is our data on a few additional immunological cell types of interest for review:

- Line 402: Sirolimus is a treatment, so author should be careful of the words used to explain Sirolimus as an inhibited upstream regulator. All regulators not linked with transcription factors should have been excluded from their upstream analyses.

We modified our wording about sirolimus:

“Among the multiple findings, interferon signaling was an activated canonical pathway. Regarding inhibition, genes that would be found downstream of the drug sirolimus were inhibited in AIH patients.”

- Line 405: conclusions made by the authors would need further supports from their data. RICTOR changes of expressions in all their samples? Predicted target changes of expression?

We modified our wording about RICTOR in the Discussion:

“RPTOR Independent Companion Of MTOR Complex 2 (RICTOR) was determined by pathway analysis to be a significantly inhibited upstream regulator of gene expression in treatment-naïve AIH patients, with multiple target molecules supporting this in the dataset. Sirolimus is a macrolide drug that inhibits mTOR, a protein that controls the proliferation and survival of activated lymphocytes. Indeed, mTOR signaling was one of the most significant pathways in our focused analysis of treatment-naïve patients vs. healthy controls (Figure S3).”

- Line 476 and 477: those lines should be removed from the discussion as theire is no correlation between pegivirus presence and AIH.

We modified the wording on pegivirus and AIH.

“We also uncovered a number of AIH samples with viral sequences, an unexpected finding that raises the possibility of molecular mimicry after viral infection as a mechanism in AIH.”

Reviewer #3: - The authors performed RNAseq in the peripheral blood of patients with auto immune hepatitis (AIH) to gain insight into the pathogenesis of this disease.

- This study is very exciting and in my opinion, figure 2A is on of the most interesting finding of the study. It compares the top 10 canonial pathways in patients with AIH vs healthy subjects. One limitation is that all AIH patients are included regardless of the treatment they received, and this can of course interfere with the pathways and be a confounding factor. When the authors perform this analysis only on patients naive of treatment (n=5), they can only draw a conclusion on interferon signaling and not on the other caonical pathways due to lack of power.

- Table 1: Treatment type. I would like the authors to give more detail (in the table or in the text) about treatment type. Treatment received is a main confounding factor in this study, therefore, it is important to have maximum information about treatment received.

-> "Other treatment": I would like to know the name of these treatments

We added information on “other treatments” to the text.

-> "Off treatment": cause ? (remission? Non observance ? )

We added information as to why certain patients were “off treatment” to the text.

-> " Steroids". Usually, AIH treatment consists of the association of steroids AND another drug (usually azathioprine). Patients are not often treated with steroids only. I would like more information about the treatment associated with steroids (if any) for each of the 28 patients on steroids.

We clarified that “steroids” indicates patients were on an AIH treatment regimen that included corticosteroids at any dose.

“10 AIH patients were off therapy at the time of blood biospecimen collection (5 were treatment-naïve, 3 were in remission and had been withdrawn from therapy, and 2 had been previously treated but were off therapy at the time of sample collection). Of subjects on AIH therapy at the time of sample collection, 28 were on a regimen that included corticosteroids: 21 with and 7 without maintenance medications. 29 of the AIH patients on therapy at the time of sample collection were on a steroid-free regimen consisting only of maintenance medications such as azathioprine, mycophenolate, or tacrolimus.”

- Figure 4: Treatment received can explain a lower CD8 count. Excluding patients on steroids (and not on other treatments that may have an impact of CD8 count such as I think azathioprine) is not enough (Figure 4 B).I think this should be mentionned in the discussion. If you want to have results more interpretable, you should repeat this analysis but for a given treatment (ie: compare count of CD8+ of patients on steroids only (or another drug but the same one), to show if there is a difference between complete and partial response).

Yes, this is an important point, we have added the following to the discussion to clarify this point. Unfortunately, we did not have a great enough sample size to perform analysis of individual subgroups by treatment type.

“Finally, dividing the AIH cohort by response to treatment and deconvoluting the gene expression data by cell type pointed to a role for CD8+ T cells. Patients with a partial response to therapy displayed a lower CD8+ T cell expression signal. CD8+ T lymphocytes are cytotoxic and induce apoptosis of damaged cells in response to antigen presentation on MHC Class I molecules, and they are a major cell type in areas of interface hepatitis.(48) In Type II AIH, the degree of CD8+ T cell response correlates with disease activity.(49) In vitro studies have shown that immunosuppressive therapy alters the ability of regulatory T cells to modulate CD8+ T cell activity (50). However, these results are limited by the fact that the effect of AIH on CD8 cell populations is difficult to truly separate from the effect of AIH therapies, eg various forms of immunosuppression. Further study is required to better understand the relative contrition of immunosuppression and underlying AIH disease activity on CD8 biology.”

- 384 "and that pegivirus is more prevalent in patients with liver transplant that hepatectomy patients" -> I do not understand what the authors means by "hepatectomy patients" (hepatectomy means the removal of one's liver).

We clarified the reference on pegivirus:

“Pegivirus is more prevalent in patients with liver transplant than partial hepatectomy patients, but pegivirus is not associated with any changes in clinical outcomes(41).”

- 432 "in many patients with early cirrhosis fibrosis stage in only apparent ...". I am not sure to understand what the authors mean by "early cirrhosis".

We clarified this sentence to read

“While some patients have obvious radiographic evidence of cirrhosis, there are many patients with early, compensated cirrhosis (i.e. without clinically evident complications), for whom fibrosis stage is only apparent on a liver biopsy.”

- 433: I would like to point out that fibroScan and shear wave devices are recognised and validated non invasive methods for the diagnosis of cirrhosis.

We acknowledged this point in the Discussion:

“Noninvasive methods of fibrosis assessment such as transient elastography and magnetic resonance elastography, while validated in AIH, are not available or accessible for many AIH patients.”

- 435 "and advanced disease". I am not sure to understand what the authors means by "advanced disease".

We clarified this to read “advanced disease (stage 3-4 fibrosis).”

- Table 2 and 3A are blurry

We have replaced these tables to make them clearer.

Attachment Submitted filename: Response to Reviewers 7-23-21.docx Click here for additional data file.

# Peer review: Decision Letter 1

**DOI:** 10.1371/journal.pone.0264307.r004

**Authors:** Gualtiero I. Colombo

9 Feb 2022

Transcriptomic Profiling of Blood from Autoimmune Hepatitis Patients Reveals Potential Mechanisms with Implications for Management

PONE-D-20-39992R1

Dear Dr. Tana,

We’re pleased to inform you that your manuscript has been judged scientifically suitable for publication and will be formally accepted for publication once it meets all outstanding technical requirements.

Within one week, you’ll receive an e-mail detailing the required amendments. When these have been addressed, you’ll receive a formal acceptance letter and your manuscript will be scheduled for publication.

An invoice for payment will follow shortly after the formal acceptance. To ensure an efficient process, please log into Editorial Manager at http://www.editorialmanager.com/pone/, click the 'Update My Information' link at the top of the page, and double check that your user information is up-to-date. If you have any billing related questions, please contact our Author Billing department directly at authorbilling@plos.org.

If your institution or institutions have a press office, please notify them about your upcoming paper to help maximize its impact. If they’ll be preparing press materials, please inform our press team as soon as possible -- no later than 48 hours after receiving the formal acceptance. Your manuscript will remain under strict press embargo until 2 pm Eastern Time on the date of publication. For more information, please contact onepress@plos.org.

Kind regards,

Gualtiero I. Colombo, M.D., Ph.D.

Academic Editor

PLOS ONE

Additional Editor Comments (optional):

Reviewers' comments:

Reviewer's Responses to Questions

**Comments to the Author**

1. If the authors have adequately addressed your comments raised in a previous round of review and you feel that this manuscript is now acceptable for publication, you may indicate that here to bypass the “Comments to the Author” section, enter your conflict of interest statement in the “Confidential to Editor” section, and submit your "Accept" recommendation.

Reviewer #1: All comments have been addressed

Reviewer #3: All comments have been addressed

**********

2. Is the manuscript technically sound, and do the data support the conclusions?

The manuscript must describe a technically sound piece of scientific research with data that supports the conclusions. Experiments must have been conducted rigorously, with appropriate controls, replication, and sample sizes. The conclusions must be drawn appropriately based on the data presented. 

Reviewer #1: Yes

Reviewer #3: Yes

**********

3. Has the statistical analysis been performed appropriately and rigorously? 

Reviewer #1: Yes

Reviewer #3: Yes

**********

4. Have the authors made all data underlying the findings in their manuscript fully available?

The PLOS Data policy requires authors to make all data underlying the findings described in their manuscript fully available without restriction, with rare exception (please refer to the Data Availability Statement in the manuscript PDF file). The data should be provided as part of the manuscript or its supporting information, or deposited to a public repository. For example, in addition to summary statistics, the data points behind means, medians and variance measures should be available. If there are restrictions on publicly sharing data—e.g. participant privacy or use of data from a third party—those must be specified.

Reviewer #1: Yes

Reviewer #3: Yes

**********

5. Is the manuscript presented in an intelligible fashion and written in standard English?

PLOS ONE does not copyedit accepted manuscripts, so the language in submitted articles must be clear, correct, and unambiguous. Any typographical or grammatical errors should be corrected at revision, so please note any specific errors here.

Reviewer #1: Yes

Reviewer #3: Yes

**********

6. Review Comments to the Author

Please use the space provided to explain your answers to the questions above. You may also include additional comments for the author, including concerns about dual publication, research ethics, or publication ethics. (Please upload your review as an attachment if it exceeds 20,000 characters)

Reviewer #1: (No Response)

Reviewer #3: All previous comments have been adressed.

This study is very interesting and exciting and I hope that it is a first step to a bigger study adressing the same questions.

**********

7. PLOS authors have the option to publish the peer review history of their article (what does this mean?). If published, this will include your full peer review and any attached files.

If you choose “no”, your identity will remain anonymous but your review may still be made public.

**Do you want your identity to be public for this peer review?** For information about this choice, including consent withdrawal, please see our Privacy Policy.

Reviewer #1: No

Reviewer #3: **Yes: **Dr Claire Mayer

# Editor report: Acceptance letter

**DOI:** 10.1371/journal.pone.0264307.r005

**Authors:** Gualtiero I. Colombo

11 Mar 2022

PONE-D-20-39992R1 

Transcriptomic Profiling of Blood from Autoimmune Hepatitis Patients Reveals Potential Mechanisms with Implications for Management 

Dear Dr. Tana:

I'm pleased to inform you that your manuscript has been deemed suitable for publication in PLOS ONE. Congratulations! Your manuscript is now with our production department. 

If your institution or institutions have a press office, please let them know about your upcoming paper now to help maximize its impact. If they'll be preparing press materials, please inform our press team within the next 48 hours. Your manuscript will remain under strict press embargo until 2 pm Eastern Time on the date of publication. For more information please contact onepress@plos.org.

If we can help with anything else, please email us at plosone@plos.org. 

Thank you for submitting your work to PLOS ONE and supporting open access. 

Kind regards, 

PLOS ONE Editorial Office Staff

on behalf of

Dr. Gualtiero I. Colombo 

Academic Editor

PLOS ONE
